# Direct top-quark decay width measurement in the $\mathbf {\varvec{t\bar{t}}}$ lepton+jets channel at $\sqrt{s}=8~\text {TeV}$ with the ATLAS experiment

**DOI:** 10.1140/epjc/s10052-018-5595-5

**Published:** 2018-02-15

**Authors:** M. Aaboud, G. Aad, B. Abbott, O. Abdinov, B. Abeloos, S. H. Abidi, O. S. AbouZeid, N. L. Abraham, H. Abramowicz, H. Abreu, R. Abreu, Y. Abulaiti, B. S. Acharya, S. Adachi, L. Adamczyk, J. Adelman, M. Adersberger, T. Adye, A. A. Affolder, Y. Afik, T. Agatonovic-Jovin, C. Agheorghiesei, J. A. Aguilar-Saavedra, S. P. Ahlen, F. Ahmadov, G. Aielli, S. Akatsuka, H. Akerstedt, T. P. A. Åkesson, E. Akilli, A. V. Akimov, G. L. Alberghi, J. Albert, P. Albicocco, M. J. Alconada Verzini, S. C. Alderweireldt, M. Aleksa, I. N. Aleksandrov, C. Alexa, G. Alexander, T. Alexopoulos, M. Alhroob, B. Ali, M. Aliev, G. Alimonti, J. Alison, S. P. Alkire, B. M. M. Allbrooke, B. W. Allen, P. P. Allport, A. Aloisio, A. Alonso, F. Alonso, C. Alpigiani, A. A. Alshehri, M. I. Alstaty, B. Alvarez Gonzalez, D. Álvarez Piqueras, M. G. Alviggi, B. T. Amadio, Y. Amaral Coutinho, C. Amelung, D. Amidei, S. P. Amor Dos Santos, S. Amoroso, G. Amundsen, C. Anastopoulos, L. S. Ancu, N. Andari, T. Andeen, C. F. Anders, J. K. Anders, K. J. Anderson, A. Andreazza, V. Andrei, S. Angelidakis, I. Angelozzi, A. Angerami, A. V. Anisenkov, N. Anjos, A. Annovi, C. Antel, M. Antonelli, A. Antonov, D. J. Antrim, F. Anulli, M. Aoki, L. Aperio Bella, G. Arabidze, Y. Arai, J. P. Araque, V. Araujo Ferraz, A. T. H. Arce, R. E. Ardell, F. A. Arduh, J.-F. Arguin, S. Argyropoulos, M. Arik, A. J. Armbruster, L. J. Armitage, O. Arnaez, H. Arnold, M. Arratia, O. Arslan, A. Artamonov, G. Artoni, S. Artz, S. Asai, N. Asbah, A. Ashkenazi, L. Asquith, K. Assamagan, R. Astalos, M. Atkinson, N. B. Atlay, K. Augsten, G. Avolio, B. Axen, M. K. Ayoub, G. Azuelos, A. E. Baas, M. J. Baca, H. Bachacou, K. Bachas, M. Backes, P. Bagnaia, M. Bahmani, H. Bahrasemani, J. T. Baines, M. Bajic, O. K. Baker, P. J. Bakker, E. M. Baldin, P. Balek, F. Balli, W. K. Balunas, E. Banas, A. Bandyopadhyay, Sw. Banerjee, A. A. E. Bannoura, L. Barak, E. L. Barberio, D. Barberis, M. Barbero, T. Barillari, M.-S. Barisits, J. T. Barkeloo, T. Barklow, N. Barlow, S. L. Barnes, B. M. Barnett, R. M. Barnett, Z. Barnovska-Blenessy, A. Baroncelli, G. Barone, A. J. Barr, L. Barranco Navarro, F. Barreiro, J. Barreiro Guimarães da Costa, R. Bartoldus, A. E. Barton, P. Bartos, A. Basalaev, A. Bassalat, R. L. Bates, S. J. Batista, J. R. Batley, M. Battaglia, M. Bauce, F. Bauer, H. S. Bawa, J. B. Beacham, M. D. Beattie, T. Beau, P. H. Beauchemin, P. Bechtle, H. P. Beck, H. C. Beck, K. Becker, M. Becker, C. Becot, A. J. Beddall, A. Beddall, V. A. Bednyakov, M. Bedognetti, C. P. Bee, T. A. Beermann, M. Begalli, M. Begel, J. K. Behr, A. S. Bell, G. Bella, L. Bellagamba, A. Bellerive, M. Bellomo, K. Belotskiy, O. Beltramello, N. L. Belyaev, O. Benary, D. Benchekroun, M. Bender, N. Benekos, Y. Benhammou, E. Benhar Noccioli, J. Benitez, D. P. Benjamin, M. Benoit, J. R. Bensinger, S. Bentvelsen, L. Beresford, M. Beretta, D. Berge, E. Bergeaas Kuutmann, N. Berger, L. J. Bergsten, J. Beringer, S. Berlendis, N. R. Bernard, G. Bernardi, C. Bernius, F. U. Bernlochner, T. Berry, P. Berta, C. Bertella, G. Bertoli, I. A. Bertram, C. Bertsche, G. J. Besjes, O. Bessidskaia Bylund, M. Bessner, N. Besson, A. Bethani, S. Bethke, A. Betti, A. J. Bevan, J. Beyer, R. M. Bianchi, O. Biebel, D. Biedermann, R. Bielski, K. Bierwagen, N. V. Biesuz, M. Biglietti, T. R. V. Billoud, H. Bilokon, M. Bindi, A. Bingul, C. Bini, S. Biondi, T. Bisanz, C. Bittrich, D. M. Bjergaard, J. E. Black, K. M. Black, R. E. Blair, T. Blazek, I. Bloch, C. Blocker, A. Blue, U. Blumenschein, S. Blunier, G. J. Bobbink, V. S. Bobrovnikov, S. S. Bocchetta, A. Bocci, C. Bock, M. Boehler, D. Boerner, D. Bogavac, A. G. Bogdanchikov, C. Bohm, V. Boisvert, P. Bokan, T. Bold, A. S. Boldyrev, A. E. Bolz, M. Bomben, M. Bona, M. Boonekamp, A. Borisov, G. Borissov, J. Bortfeldt, D. Bortoletto, V. Bortolotto, D. Boscherini, M. Bosman, J. D. Bossio Sola, J. Boudreau, E. V. Bouhova-Thacker, D. Boumediene, C. Bourdarios, S. K. Boutle, A. Boveia, J. Boyd, I. R. Boyko, A. J. Bozson, J. Bracinik, A. Brandt, G. Brandt, O. Brandt, F. Braren, U. Bratzler, B. Brau, J. E. Brau, W. D. Breaden Madden, K. Brendlinger, A. J. Brennan, L. Brenner, R. Brenner, S. Bressler, D. L. Briglin, T. M. Bristow, D. Britton, D. Britzger, F. M. Brochu, I. Brock, R. Brock, G. Brooijmans, T. Brooks, W. K. Brooks, J. Brosamer, E. Brost, J. H Broughton, P. A. Bruckman de Renstrom, D. Bruncko, A. Bruni, G. Bruni, L. S. Bruni, S. Bruno, BH Brunt, M. Bruschi, N. Bruscino, P. Bryant, L. Bryngemark, T. Buanes, Q. Buat, P. Buchholz, A. G. Buckley, I. A. Budagov, F. Buehrer, M. K. Bugge, O. Bulekov, D. Bullock, T. J. Burch, S. Burdin, C. D. Burgard, A. M. Burger, B. Burghgrave, K. Burka, S. Burke, I. Burmeister, J. T. P. Burr, D. Büscher, V. Büscher, P. Bussey, J. M. Butler, C. M. Buttar, J. M. Butterworth, P. Butti, W. Buttinger, A. Buzatu, A. R. Buzykaev, S. Cabrera Urbán, D. Caforio, H. Cai, V. M. Cairo, O. Cakir, N. Calace, P. Calafiura, A. Calandri, G. Calderini, P. Calfayan, G. Callea, L. P. Caloba, S. Calvente Lopez, D. Calvet, S. Calvet, T. P. Calvet, R. Camacho Toro, S. Camarda, P. Camarri, D. Cameron, R. Caminal Armadans, C. Camincher, S. Campana, M. Campanelli, A. Camplani, A. Campoverde, V. Canale, M. Cano Bret, J. Cantero, T. Cao, M. D. M. Capeans Garrido, I. Caprini, M. Caprini, M. Capua, R. M. Carbone, R. Cardarelli, F. Cardillo, I. Carli, T. Carli, G. Carlino, B. T. Carlson, L. Carminati, R. M. D. Carney, S. Caron, E. Carquin, S. Carrá, G. D. Carrillo-Montoya, D. Casadei, M. P. Casado, D. W. Casper, R. Castelijn, V. Castillo Gimenez, N. F. Castro, A. Catinaccio, J. R. Catmore, A. Cattai, J. Caudron, V. Cavaliere, E. Cavallaro, D. Cavalli, M. Cavalli-Sforza, V. Cavasinni, E. Celebi, F. Ceradini, L. Cerda Alberich, A. S. Cerqueira, A. Cerri, L. Cerrito, F. Cerutti, A. Cervelli, S. A. Cetin, A. Chafaq, D. Chakraborty, S. K. Chan, W. S. Chan, Y. L. Chan, P. Chang, J. D. Chapman, D. G. Charlton, C. C. Chau, C. A. Chavez Barajas, S. Che, S. Cheatham, A. Chegwidden, S. Chekanov, S. V. Chekulaev, G. A. Chelkov, M. A. Chelstowska, C. Chen, C. Chen, H. Chen, J. Chen, S. Chen, S. Chen, X. Chen, Y. Chen, H. C. Cheng, H. J. Cheng, A. Cheplakov, E. Cheremushkina, R. Cherkaoui El Moursli, E. Cheu, K. Cheung, L. Chevalier, V. Chiarella, G. Chiarelli, G. Chiodini, A. S. Chisholm, A. Chitan, Y. H. Chiu, M. V. Chizhov, K. Choi, A. R. Chomont, S. Chouridou, Y. S. Chow, V. Christodoulou, M. C. Chu, J. Chudoba, A. J. Chuinard, J. J. Chwastowski, L. Chytka, A. K. Ciftci, D. Cinca, V. Cindro, I. A. Cioara, A. Ciocio, F. Cirotto, Z. H. Citron, M. Citterio, M. Ciubancan, A. Clark, B. L. Clark, M. R. Clark, P. J. Clark, R. N. Clarke, C. Clement, Y. Coadou, M. Cobal, A. Coccaro, J. Cochran, L. Colasurdo, B. Cole, A. P. Colijn, J. Collot, T. Colombo, P. Conde Muiño, E. Coniavitis, S. H. Connell, I. A. Connelly, S. Constantinescu, G. Conti, F. Conventi, M. Cooke, A. M. Cooper-Sarkar, F. Cormier, K. J. R. Cormier, M. Corradi, F. Corriveau, A. Cortes-Gonzalez, G. Costa, M. J. Costa, D. Costanzo, G. Cottin, G. Cowan, B. E. Cox, K. Cranmer, S. J. Crawley, R. A. Creager, G. Cree, S. Crépé-Renaudin, F. Crescioli, W. A. Cribbs, M. Cristinziani, V. Croft, G. Crosetti, A. Cueto, T. Cuhadar Donszelmann, A. R. Cukierman, J. Cummings, M. Curatolo, J. Cúth, S. Czekierda, P. Czodrowski, G. D’amen, S. D’Auria, L. D’eramo, M. D’Onofrio, M. J. Da Cunha Sargedas De Sousa, C. Da Via, W. Dabrowski, T. Dado, T. Dai, O. Dale, F. Dallaire, C. Dallapiccola, M. Dam, J. R. Dandoy, M. F. Daneri, N. P. Dang, A. C. Daniells, N. S. Dann, M. Danninger, M. Dano Hoffmann, V. Dao, G. Darbo, S. Darmora, J. Dassoulas, A. Dattagupta, T. Daubney, W. Davey, C. David, T. Davidek, D. R. Davis, P. Davison, E. Dawe, I. Dawson, K. De, R. de Asmundis, A. De Benedetti, S. De Castro, S. De Cecco, N. De Groot, P. de Jong, H. De la Torre, F. De Lorenzi, A. De Maria, D. De Pedis, A. De Salvo, U. De Sanctis, A. De Santo, K. De Vasconcelos Corga, J. B. De Vivie DeRegie, R. Debbe, C. Debenedetti, D. V. Dedovich, N. Dehghanian, I. Deigaard, M. Del Gaudio, J. Del Peso, D. Delgove, F. Deliot, C. M. Delitzsch, A. Dell’Acqua, L. Dell’Asta, M. Dell’Orso, M. Della Pietra, D. della Volpe, M. Delmastro, C. Delporte, P. A. Delsart, D. A. DeMarco, S. Demers, M. Demichev, A. Demilly, S. P. Denisov, D. Denysiuk, D. Derendarz, J. E. Derkaoui, F. Derue, P. Dervan, K. Desch, C. Deterre, K. Dette, M. R. Devesa, P. O. Deviveiros, A. Dewhurst, S. Dhaliwal, F. A. Di Bello, A. Di Ciaccio, L. Di Ciaccio, W. K. Di Clemente, C. Di Donato, A. Di Girolamo, B. Di Girolamo, B. Di Micco, R. Di Nardo, K. F. Di Petrillo, A. Di Simone, R. Di Sipio, D. Di Valentino, C. Diaconu, M. Diamond, F. A. Dias, M. A. Diaz, E. B. Diehl, J. Dietrich, S. Díez Cornell, A. Dimitrievska, J. Dingfelder, P. Dita, S. Dita, F. Dittus, F. Djama, T. Djobava, J. I. Djuvsland, M. A. B. do Vale, D. Dobos, M. Dobre, D. Dodsworth, C. Doglioni, J. Dolejsi, Z. Dolezal, M. Donadelli, S. Donati, P. Dondero, J. Donini, J. Dopke, A. Doria, M. T. Dova, A. T. Doyle, E. Drechsler, M. Dris, Y. Du, J. Duarte-Campderros, F. Dubinin, A. Dubreuil, E. Duchovni, G. Duckeck, A. Ducourthial, O. A. Ducu, D. Duda, A. Dudarev, A. Chr. Dudder, E. M. Duffield, L. Duflot, M. Dührssen, C. Dulsen, M. Dumancic, A. E. Dumitriu, A. K. Duncan, M. Dunford, A. Duperrin, H. DuranYildiz, M. Düren, A. Durglishvili, D. Duschinger, B. Dutta, D. Duvnjak, M. Dyndal, B. S. Dziedzic, C. Eckardt, K. M. Ecker, R. C. Edgar, T. Eifert, G. Eigen, K. Einsweiler, T. Ekelof, M. El Kacimi, R. El Kosseifi, V. Ellajosyula, M. Ellert, S. Elles, F. Ellinghaus, A. A. Elliot, N. Ellis, J. Elmsheuser, M. Elsing, D. Emeliyanov, Y. Enari, J. S. Ennis, M. B. Epland, J. Erdmann, A. Ereditato, M. Ernst, S. Errede, M. Escalier, C. Escobar, B. Esposito, O. Estrada Pastor, A. I. Etienvre, E. Etzion, H. Evans, A. Ezhilov, M. Ezzi, F. Fabbri, L. Fabbri, V. Fabiani, G. Facini, R. M. Fakhrutdinov, S. Falciano, R. J. Falla, J. Faltova, Y. Fang, M. Fanti, A. Farbin, A. Farilla, C. Farina, E. M. Farina, T. Farooque, S. Farrell, S. M. Farrington, P. Farthouat, F. Fassi, P. Fassnacht, D. Fassouliotis, M. Faucci Giannelli, A. Favareto, W. J. Fawcett, L. Fayard, O. L. Fedin, W. Fedorko, S. Feigl, L. Feligioni, C. Feng, E. J. Feng, M. J. Fenton, A. B. Fenyuk, L. Feremenga, P. Fernandez Martinez, J. Ferrando, A. Ferrari, P. Ferrari, R. Ferrari, D. E. Ferreira de Lima, A. Ferrer, D. Ferrere, C. Ferretti, F. Fiedler, A. Filipčič, M. Filipuzzi, F. Filthaut, M. Fincke-Keeler, K. D. Finelli, M. C. N. Fiolhais, L. Fiorini, A. Fischer, C. Fischer, J. Fischer, W. C. Fisher, N. Flaschel, I. Fleck, P. Fleischmann, R. R. M. Fletcher, T. Flick, B. M. Flierl, L. R. Flores Castillo, M. J. Flowerdew, G. T. Forcolin, A. Formica, F. A. Förster, A. Forti, A. G. Foster, D. Fournier, H. Fox, S. Fracchia, P. Francavilla, M. Franchini, S. Franchino, D. Francis, L. Franconi, M. Franklin, M. Frate, M. Fraternali, D. Freeborn, S. M. Fressard-Batraneanu, B. Freund, D. Froidevaux, J. A. Frost, C. Fukunaga, T. Fusayasu, J. Fuster, O. Gabizon, A. Gabrielli, A. Gabrielli, G. P. Gach, S. Gadatsch, S. Gadomski, G. Gagliardi, L. G. Gagnon, C. Galea, B. Galhardo, E. J. Gallas, B. J. Gallop, P. Gallus, G. Galster, K. K. Gan, S. Ganguly, Y. Gao, Y. S. Gao, F. M. GarayWalls, C. García, J. E. GarcíaNavarro, J. A. GarcíaPascual, M. Garcia-Sciveres, R. W. Gardner, N. Garelli, V. Garonne, A. Gascon Bravo, K. Gasnikova, C. Gatti, A. Gaudiello, G. Gaudio, I. L. Gavrilenko, C. Gay, G. Gaycken, E. N. Gazis, C. N. P. Gee, J. Geisen, M. Geisen, M. P. Geisler, K. Gellerstedt, C. Gemme, M. H. Genest, C. Geng, S. Gentile, C. Gentsos, S. George, D. Gerbaudo, G. Geßner, S. Ghasemi, M. Ghneimat, B. Giacobbe, S. Giagu, N. Giangiacomi, P. Giannetti, S. M. Gibson, M. Gignac, M. Gilchriese, D. Gillberg, G. Gilles, D. M. Gingrich, M. P. Giordani, F. M. Giorgi, P. F. Giraud, P. Giromini, G. Giugliarelli, D. Giugni, F. Giuli, C. Giuliani, M. Giulini, B. K. Gjelsten, S. Gkaitatzis, I. Gkialas, E. L. Gkougkousis, P. Gkountoumis, L. K. Gladilin, C. Glasman, J. Glatzer, P. C. F. Glaysher, A. Glazov, M. Goblirsch-Kolb, J. Godlewski, S. Goldfarb, T. Golling, D. Golubkov, A. Gomes, R. Gonçalo, R. Goncalves Gama, J. Goncalves Pinto Firmino Da Costa, G. Gonella, L. Gonella, A. Gongadze, J. L. Gonski, S. González de la Hoz, S. Gonzalez-Sevilla, L. Goossens, P. A. Gorbounov, H. A. Gordon, I. Gorelov, B. Gorini, E. Gorini, A. Gorišek, A. T. Goshaw, C. Gössling, M. I. Gostkin, C. A. Gottardo, C. R. Goudet, D. Goujdami, A. G. Goussiou, N. Govender, E. Gozani, I. Grabowska-Bold, P. O. J. Gradin, J. Gramling, E. Gramstad, S. Grancagnolo, V. Gratchev, P. M. Gravila, C. Gray, H. M. Gray, Z. D. Greenwood, C. Grefe, K. Gregersen, I. M. Gregor, P. Grenier, K. Grevtsov, J. Griffiths, A. A. Grillo, K. Grimm, S. Grinstein, Ph. Gris, J.-F. Grivaz, S. Groh, E. Gross, J. Grosse-Knetter, G. C. Grossi, Z. J. Grout, A. Grummer, L. Guan, W. Guan, J. Guenther, F. Guescini, D. Guest, O. Gueta, B. Gui, E. Guido, T. Guillemin, S. Guindon, U. Gul, C. Gumpert, J. Guo, W. Guo, Y. Guo, R. Gupta, S. Gurbuz, G. Gustavino, B. J. Gutelman, P. Gutierrez, N. G. Gutierrez Ortiz, C. Gutschow, C. Guyot, M. P. Guzik, C. Gwenlan, C. B. Gwilliam, A. Haas, C. Haber, H. K. Hadavand, N. Haddad, A. Hadef, S. Hageböck, M. Hagihara, H. Hakobyan, M. Haleem, J. Haley, G. Halladjian, G. D. Hallewell, K. Hamacher, P. Hamal, K. Hamano, A. Hamilton, G. N. Hamity, P. G. Hamnett, L. Han, S. Han, K. Hanagaki, K. Hanawa, M. Hance, D. M. Handl, B. Haney, P. Hanke, J. B. Hansen, J. D. Hansen, M. C. Hansen, P. H. Hansen, K. Hara, A. S. Hard, T. Harenberg, F. Hariri, S. Harkusha, P. F. Harrison, N. M. Hartmann, Y. Hasegawa, A. Hasib, S. Hassani, S. Haug, R. Hauser, L. Hauswald, L. B. Havener, M. Havranek, C. M. Hawkes, R. J. Hawkings, D. Hayakawa, D. Hayden, C. P. Hays, J. M. Hays, H. S. Hayward, S. J. Haywood, S. J. Head, T. Heck, V. Hedberg, L. Heelan, S. Heer, K. K. Heidegger, S. Heim, T. Heim, B. Heinemann, J. J. Heinrich, L. Heinrich, C. Heinz, J. Hejbal, L. Helary, A. Held, S. Hellman, C. Helsens, R. C. W. Henderson, Y. Heng, S. Henkelmann, A. M. Henriques Correia, S. Henrot-Versille, G. H. Herbert, H. Herde, V. Herget, Y. Hernández Jiménez, H. Herr, G. Herten, R. Hertenberger, L. Hervas, T. C. Herwig, G. G. Hesketh, N. P. Hessey, J. W. Hetherly, S. Higashino, E. Higón-Rodriguez, K. Hildebrand, E. Hill, J. C. Hill, K. H. Hiller, S. J. Hillier, M. Hils, I. Hinchliffe, M. Hirose, D. Hirschbuehl, B. Hiti, O. Hladik, D. R. Hlaluku, X. Hoad, J. Hobbs, N. Hod, M. C. Hodgkinson, P. Hodgson, A. Hoecker, M. R. Hoeferkamp, F. Hoenig, D. Hohn, T. R. Holmes, M. Homann, S. Honda, T. Honda, T. M. Hong, B. H. Hooberman, W. H. Hopkins, Y. Horii, A. J. Horton, J.-Y. Hostachy, A. Hostiuc, S. Hou, A. Hoummada, J. Howarth, J. Hoya, M. Hrabovsky, J. Hrdinka, I. Hristova, J. Hrivnac, T. Hryn’ova, A. Hrynevich, P. J. Hsu, S.-C. Hsu, Q. Hu, S. Hu, Y. Huang, Z. Hubacek, F. Hubaut, F. Huegging, T. B. Huffman, E. W. Hughes, M. Huhtinen, R. F. H. Hunter, P. Huo, N. Huseynov, J. Huston, J. Huth, R. Hyneman, G. Iacobucci, G. Iakovidis, I. Ibragimov, L. Iconomidou-Fayard, Z. Idrissi, P. Iengo, O. Igonkina, T. Iizawa, Y. Ikegami, M. Ikeno, Y. Ilchenko, D. Iliadis, N. Ilic, F. Iltzsche, G. Introzzi, P. Ioannou, M. Iodice, K. Iordanidou, V. Ippolito, M. F. Isacson, N. Ishijima, M. Ishino, M. Ishitsuka, C. Issever, S. Istin, F. Ito, J. M. IturbePonce, R. Iuppa, H. Iwasaki, J. M. Izen, V. Izzo, S. Jabbar, P. Jackson, R. M. Jacobs, V. Jain, K. B. Jakobi, K. Jakobs, S. Jakobsen, T. Jakoubek, D. O. Jamin, D. K. Jana, R. Jansky, J. Janssen, M. Janus, P. A. Janus, G. Jarlskog, N. Javadov, T. Javůrek, M. Javurkova, F. Jeanneau, L. Jeanty, J. Jejelava, A. Jelinskas, P. Jenni, C. Jeske, S. Jézéquel, H. Ji, J. Jia, H. Jiang, Y. Jiang, Z. Jiang, S. Jiggins, J. Jimenez Pena, S. Jin, A. Jinaru, O. Jinnouchi, H. Jivan, P. Johansson, K. A. Johns, C. A. Johnson, W. J. Johnson, K. Jon-And, R. W. L. Jones, S. D. Jones, S. Jones, T. J. Jones, J. Jongmanns, P. M. Jorge, J. Jovicevic, X. Ju, A. Juste Rozas, M. K. Köhler, A. Kaczmarska, M. Kado, H. Kagan, M. Kagan, S. J. Kahn, T. Kaji, E. Kajomovitz, C. W. Kalderon, A. Kaluza, S. Kama, A. Kamenshchikov, N. Kanaya, L. Kanjir, V. A. Kantserov, J. Kanzaki, B. Kaplan, L. S. Kaplan, D. Kar, K. Karakostas, N. Karastathis, M. J. Kareem, E. Karentzos, S. N. Karpov, Z. M. Karpova, K. Karthik, V. Kartvelishvili, A. N. Karyukhin, K. Kasahara, L. Kashif, R. D. Kass, A. Kastanas, Y. Kataoka, C. Kato, A. Katre, J. Katzy, K. Kawade, K. Kawagoe, T. Kawamoto, G. Kawamura, E. F. Kay, V. F. Kazanin, R. Keeler, R. Kehoe, J. S. Keller, E. Kellermann, J. J. Kempster, J Kendrick, H. Keoshkerian, O. Kepka, B. P. Kerševan, S. Kersten, R. A. Keyes, M. Khader, F. Khalil-zada, A. Khanov, A. G. Kharlamov, T. Kharlamova, A. Khodinov, T. J. Khoo, V. Khovanskiy, E. Khramov, J. Khubua, S. Kido, C. R. Kilby, H. Y. Kim, S. H. Kim, Y. K. Kim, N. Kimura, O. M. Kind, B. T. King, D. Kirchmeier, J. Kirk, A. E. Kiryunin, T. Kishimoto, D. Kisielewska, V. Kitali, O. Kivernyk, E. Kladiva, T. Klapdor-Kleingrothaus, M. H. Klein, M. Klein, U. Klein, K. Kleinknecht, P. Klimek, A. Klimentov, R. Klingenberg, T. Klingl, T. Klioutchnikova, F. F. Klitzner, E.-E. Kluge, P. Kluit, S. Kluth, E. Kneringer, E. B. F. G. Knoops, A. Knue, A. Kobayashi, D. Kobayashi, T. Kobayashi, M. Kobel, M. Kocian, P. Kodys, T. Koffas, E. Koffeman, N. M. Köhler, T. Koi, M. Kolb, I. Koletsou, A. A. Komar, T. Kondo, N. Kondrashova, K. Köneke, A. C. König, T. Kono, R. Konoplich, N. Konstantinidis, B. Konya, R. Kopeliansky, S. Koperny, A. K. Kopp, K. Korcyl, K. Kordas, A. Korn, A. A. Korol, I. Korolkov, E. V. Korolkova, O. Kortner, S. Kortner, T. Kosek, V. V. Kostyukhin, A. Kotwal, A. Koulouris, A. Kourkoumeli-Charalampidi, C. Kourkoumelis, E. Kourlitis, V. Kouskoura, A. B. Kowalewska, R. Kowalewski, T. Z. Kowalski, C. Kozakai, W. Kozanecki, A. S. Kozhin, V. A. Kramarenko, G. Kramberger, D. Krasnopevtsev, M. W. Krasny, A. Krasznahorkay, D. Krauss, J. A. Kremer, J. Kretzschmar, K. Kreutzfeldt, P. Krieger, K. Krizka, K. Kroeninger, H. Kroha, J. Kroll, J. Kroll, J. Kroseberg, J. Krstic, U. Kruchonak, H. Krüger, N. Krumnack, M. C. Kruse, T. Kubota, H. Kucuk, S. Kuday, J. T. Kuechler, S. Kuehn, A. Kugel, F. Kuger, T. Kuhl, V. Kukhtin, R. Kukla, Y. Kulchitsky, S. Kuleshov, Y. P. Kulinich, M. Kuna, T. Kunigo, A. Kupco, T. Kupfer, O. Kuprash, H. Kurashige, L. L. Kurchaninov, Y. A. Kurochkin, M. G. Kurth, E. S. Kuwertz, M. Kuze, J. Kvita, T. Kwan, D. Kyriazopoulos, A. La Rosa, J. L. La RosaNavarro, L. La Rotonda, F. La Ruffa, C. Lacasta, F. Lacava, J. Lacey, D. P. J. Lack, H. Lacker, D. Lacour, E. Ladygin, R. Lafaye, B. Laforge, T. Lagouri, S. Lai, S. Lammers, W. Lampl, E. Lançon, U. Landgraf, M. P. J. Landon, M. C. Lanfermann, V. S. Lang, J. C. Lange, R. J. Langenberg, A. J. Lankford, F. Lanni, K. Lantzsch, A. Lanza, A. Lapertosa, S. Laplace, J. F. Laporte, T. Lari, F. Lasagni Manghi, M. Lassnig, T. S. Lau, P. Laurelli, W. Lavrijsen, A. T. Law, P. Laycock, T. Lazovich, M. Lazzaroni, B. Le, O. LeDortz, E. Le Guirriec, E. P. Le Quilleuc, M. LeBlanc, T. LeCompte, F. Ledroit-Guillon, C. A. Lee, G. R. Lee, S. C. Lee, L. Lee, B. Lefebvre, G. Lefebvre, M. Lefebvre, F. Legger, C. Leggett, G. Lehmann Miotto, X. Lei, W. A. Leight, M. A. L. Leite, R. Leitner, D. Lellouch, B. Lemmer, K. J. C. Leney, T. Lenz, B. Lenzi, R. Leone, S. Leone, C. Leonidopoulos, G. Lerner, C. Leroy, R. Les, A. A. J. Lesage, C. G. Lester, M. Levchenko, J. Levêque, D. Levin, L. J. Levinson, M. Levy, D. Lewis, B. Li, Changqiao Li, H. Li, L. Li, Q. Li, Q. Li, S. Li, X. Li, Y. Li, Z. Liang, B. Liberti, A. Liblong, K. Lie, J. Liebal, W. Liebig, A. Limosani, C. Y. Lin, K. Lin, S. C. Lin, T. H. Lin, R. A. Linck, B. E. Lindquist, A. E. Lionti, E. Lipeles, A. Lipniacka, M. Lisovyi, T. M. Liss, A. Lister, A. M. Litke, B. Liu, H. Liu, H. Liu, J. K. K. Liu, J. Liu, J. B. Liu, K. Liu, L. Liu, M. Liu, Y. L. Liu, Y. Liu, M. Livan, A. Lleres, J. Llorente Merino, S. L. Lloyd, C. Y. Lo, F. Lo Sterzo, E. M. Lobodzinska, P. Loch, F. K. Loebinger, A. Loesle, K. M. Loew, T. Lohse, K. Lohwasser, M. Lokajicek, B. A. Long, J. D. Long, R. E. Long, L. Longo, K. A. Looper, J. A. Lopez, I. Lopez Paz, A. Lopez Solis, J. Lorenz, N. Lorenzo Martinez, M. Losada, P. J. Lösel, X. Lou, A. Lounis, J. Love, P. A. Love, H. Lu, N. Lu, Y. J. Lu, H. J. Lubatti, C. Luci, A. Lucotte, C. Luedtke, F. Luehring, W. Lukas, L. Luminari, O. Lundberg, B. Lund-Jensen, M. S. Lutz, P. M. Luzi, D. Lynn, R. Lysak, E. Lytken, F. Lyu, V. Lyubushkin, H. Ma, L. L. Ma, Y. Ma, G. Maccarrone, A. Macchiolo, C. M. Macdonald, B. Maček, J. Machado Miguens, D. Madaffari, R. Madar, W. F. Mader, A. Madsen, N. Madysa, J. Maeda, S. Maeland, T. Maeno, A. S. Maevskiy, V. Magerl, C. Maiani, C. Maidantchik, T. Maier, A. Maio, O. Majersky, S. Majewski, Y. Makida, N. Makovec, B. Malaescu, Pa. Malecki, V. P. Maleev, F. Malek, U. Mallik, D. Malon, C. Malone, S. Maltezos, S. Malyukov, J. Mamuzic, G. Mancini, I. Mandić, J. Maneira, L. Manhaes de Andrade Filho, J. Manjarres Ramos, K. H. Mankinen, A. Mann, A. Manousos, B. Mansoulie, J. D. Mansour, R. Mantifel, M. Mantoani, S. Manzoni, L. Mapelli, G. Marceca, L. March, L. Marchese, G. Marchiori, M. Marcisovsky, C. A. Marin Tobon, M. Marjanovic, D. E. Marley, F. Marroquim, S. P. Marsden, Z. Marshall, M. U. F Martensson, S. Marti-Garcia, C. B. Martin, T. A. Martin, V. J. Martin, B. Martin dit Latour, M. Martinez, V. I. Martinez Outschoorn, S. Martin-Haugh, V. S. Martoiu, A. C. Martyniuk, A. Marzin, L. Masetti, T. Mashimo, R. Mashinistov, J. Masik, A. L. Maslennikov, L. H. Mason, L. Massa, P. Mastrandrea, A. Mastroberardino, T. Masubuchi, P. Mättig, J. Maurer, S. J. Maxfield, D. A. Maximov, R. Mazini, I. Maznas, S. M. Mazza, N. C. Mc Fadden, G. Mc Goldrick, S. P. Mc Kee, A. McCarn, R. L. McCarthy, T. G. McCarthy, L. I. McClymont, E. F. McDonald, J. A. Mcfayden, G. Mchedlidze, S. J. McMahon, P. C. McNamara, C. J. McNicol, R. A. McPherson, S. Meehan, T. J. Megy, S. Mehlhase, A. Mehta, T. Meideck, K. Meier, B. Meirose, D. Melini, B. R. Mellado Garcia, J. D. Mellenthin, M. Melo, F. Meloni, A. Melzer, S. B. Menary, L. Meng, X. T. Meng, A. Mengarelli, S. Menke, E. Meoni, S. Mergelmeyer, C. Merlassino, P. Mermod, L. Merola, C. Meroni, F. S. Merritt, A. Messina, J. Metcalfe, A. S. Mete, C. Meyer, J.-P. Meyer, J. Meyer, H. Meyer Zu Theenhausen, F. Miano, R. P. Middleton, S. Miglioranzi, L. Mijović, G. Mikenberg, M. Mikestikova, M. Mikuž, M. Milesi, A. Milic, D. A. Millar, D. W. Miller, C. Mills, A. Milov, D. A. Milstead, A. A. Minaenko, Y. Minami, I. A. Minashvili, A. I. Mincer, B. Mindur, M. Mineev, Y. Minegishi, Y. Ming, L. M. Mir, A. Mirto, K. P. Mistry, T. Mitani, J. Mitrevski, V. A. Mitsou, A. Miucci, P. S. Miyagawa, A. Mizukami, J. U. Mjörnmark, T. Mkrtchyan, M. Mlynarikova, T. Moa, K. Mochizuki, P. Mogg, S. Mohapatra, S. Molander, R. Moles-Valls, M. C. Mondragon, K. Mönig, J. Monk, E. Monnier, A. Montalbano, J. Montejo Berlingen, F. Monticelli, S. Monzani, R. W. Moore, N. Morange, D. Moreno, M. Moreno Llácer, P. Morettini, S. Morgenstern, D. Mori, T. Mori, M. Morii, M. Morinaga, V. Morisbak, A. K. Morley, G. Mornacchi, J. D. Morris, L. Morvaj, P. Moschovakos, M. Mosidze, H. J. Moss, J. Moss, K. Motohashi, R. Mount, E. Mountricha, E. J. W. Moyse, S. Muanza, F. Mueller, J. Mueller, R. S. P. Mueller, D. Muenstermann, P. Mullen, G. A. Mullier, F. J. Munoz Sanchez, W. J. Murray, H. Musheghyan, M. Muškinja, A. G. Myagkov, M. Myska, B. P. Nachman, O. Nackenhorst, K. Nagai, R. Nagai, K. Nagano, Y. Nagasaka, K. Nagata, M. Nagel, E. Nagy, A. M. Nairz, Y. Nakahama, K. Nakamura, T. Nakamura, I. Nakano, R. F. Naranjo Garcia, R. Narayan, D. I. NarriasVillar, I. Naryshkin, T. Naumann, G. Navarro, R. Nayyar, H. A. Neal, P. Yu. Nechaeva, T. J. Neep, A. Negri, M. Negrini, S. Nektarijevic, C. Nellist, A. Nelson, M. E. Nelson, S. Nemecek, P. Nemethy, M. Nessi, M. S. Neubauer, M. Neumann, P. R. Newman, T. Y. Ng, Y. S. Ng, T. NguyenManh, R. B. Nickerson, R. Nicolaidou, J. Nielsen, N. Nikiforou, V. Nikolaenko, I. Nikolic-Audit, K. Nikolopoulos, P. Nilsson, Y. Ninomiya, A. Nisati, N. Nishu, R. Nisius, I. Nitsche, T. Nitta, T. Nobe, Y. Noguchi, M. Nomachi, I. Nomidis, M. A. Nomura, T. Nooney, M. Nordberg, N. Norjoharuddeen, O. Novgorodova, M. Nozaki, L. Nozka, K. Ntekas, E. Nurse, F. Nuti, K. O’connor, D. C. O’Neil, A. A. O’Rourke, V. O’Shea, F. G. Oakham, H. Oberlack, T. Obermann, J. Ocariz, A. Ochi, I. Ochoa, J. P. Ochoa-Ricoux, S. Oda, S. Odaka, A. Oh, S. H. Oh, C. C. Ohm, H. Ohman, H. Oide, H. Okawa, Y. Okumura, T. Okuyama, A. Olariu, L. F. Oleiro Seabra, S. A. Olivares Pino, D. Oliveira Damazio, M. J. R. Olsson, A. Olszewski, J. Olszowska, A. Onofre, K. Onogi, P. U. E. Onyisi, H. Oppen, M. J. Oreglia, Y. Oren, D. Orestano, N. Orlando, R. S. Orr, B. Osculati, R. Ospanov, G. Otero y Garzon, H. Otono, M. Ouchrif, F. Ould-Saada, A. Ouraou, K. P. Oussoren, Q. Ouyang, M. Owen, R. E. Owen, V. E. Ozcan, N. Ozturk, K. Pachal, A. Pacheco Pages, L. Pacheco Rodriguez, C. Padilla Aranda, S. Pagan Griso, M. Paganini, F. Paige, G. Palacino, S. Palazzo, S. Palestini, M. Palka, D. Pallin, E. St. Panagiotopoulou, I. Panagoulias, C. E. Pandini, J. G. Panduro Vazquez, P. Pani, S. Panitkin, D. Pantea, L. Paolozzi, Th. D. Papadopoulou, K. Papageorgiou, A. Paramonov, D. Paredes Hernandez, A. J. Parker, M. A. Parker, K. A. Parker, F. Parodi, J. A. Parsons, U. Parzefall, V. R. Pascuzzi, J. M. Pasner, E. Pasqualucci, S. Passaggio, Fr. Pastore, S. Pataraia, J. R. Pater, T. Pauly, B. Pearson, S. Pedraza Lopez, R. Pedro, S. V. Peleganchuk, O. Penc, C. Peng, H. Peng, J. Penwell, B. S. Peralva, M. M. Perego, D. V. Perepelitsa, F. Peri, L. Perini, H. Pernegger, S. Perrella, R. Peschke, V. D. Peshekhonov, K. Peters, R. F. Y. Peters, B. A. Petersen, T. C. Petersen, E. Petit, A. Petridis, C. Petridou, P. Petroff, E. Petrolo, M. Petrov, F. Petrucci, N. E. Pettersson, A. Peyaud, R. Pezoa, F. H. Phillips, P. W. Phillips, G. Piacquadio, E. Pianori, A. Picazio, M. A. Pickering, R. Piegaia, J. E. Pilcher, A. D. Pilkington, M. Pinamonti, J. L. Pinfold, H. Pirumov, M. Pitt, L. Plazak, M.-A. Pleier, V. Pleskot, E. Plotnikova, D. Pluth, P. Podberezko, R. Poettgen, R. Poggi, L. Poggioli, I. Pogrebnyak, D. Pohl, I. Pokharel, G. Polesello, A. Poley, A. Policicchio, R. Polifka, A. Polini, C. S. Pollard, V. Polychronakos, K. Pommès, D. Ponomarenko, L. Pontecorvo, G. A. Popeneciu, D. M. Portillo Quintero, S. Pospisil, K. Potamianos, I. N. Potrap, C. J. Potter, H. Potti, T. Poulsen, J. Poveda, M. E. PozoAstigarraga, P. Pralavorio, A. Pranko, S. Prell, D. Price, M. Primavera, S. Prince, N. Proklova, K. Prokofiev, F. Prokoshin, S. Protopopescu, J. Proudfoot, M. Przybycien, A. Puri, P. Puzo, J. Qian, G. Qin, Y. Qin, A. Quadt, M. Queitsch-Maitland, D. Quilty, S. Raddum, V. Radeka, V. Radescu, S. K. Radhakrishnan, P. Radloff, P. Rados, F. Ragusa, G. Rahal, J. A. Raine, S. Rajagopalan, C. Rangel-Smith, T. Rashid, S. Raspopov, M. G. Ratti, D. M. Rauch, F. Rauscher, S. Rave, I. Ravinovich, J. H. Rawling, M. Raymond, A. L. Read, N. P. Readioff, M. Reale, D. M. Rebuzzi, A. Redelbach, G. Redlinger, R. Reece, R. G. Reed, K. Reeves, L. Rehnisch, J. Reichert, A. Reiss, C. Rembser, H. Ren, M. Rescigno, S. Resconi, E. D. Resseguie, S. Rettie, E. Reynolds, O. L. Rezanova, P. Reznicek, R. Rezvani, R. Richter, S. Richter, E. Richter-Was, O. Ricken, M. Ridel, P. Rieck, C. J. Riegel, J. Rieger, O. Rifki, M. Rijssenbeek, A. Rimoldi, M. Rimoldi, L. Rinaldi, G. Ripellino, B. Ristić, E. Ritsch, I. Riu, F. Rizatdinova, E. Rizvi, C. Rizzi, R. T. Roberts, S. H. Robertson, A. Robichaud-Veronneau, D. Robinson, J. E. M. Robinson, A. Robson, E. Rocco, C. Roda, Y. Rodina, S. Rodriguez Bosca, A. Rodriguez Perez, D. Rodriguez Rodriguez, S. Roe, C. S. Rogan, O. Røhne, J. Roloff, A. Romaniouk, M. Romano, S. M. Romano Saez, E. Romero Adam, N. Rompotis, M. Ronzani, L. Roos, S. Rosati, K. Rosbach, P. Rose, N.-A. Rosien, E. Rossi, L. P. Rossi, J. H. N. Rosten, R. Rosten, M. Rotaru, J. Rothberg, D. Rousseau, A. Rozanov, Y. Rozen, X. Ruan, F. Rubbo, E. M. Ruettinger, F. Rühr, A. Ruiz-Martinez, Z. Rurikova, N. A. Rusakovich, H. L. Russell, J. P. Rutherfoord, N. Ruthmann, Y. F. Ryabov, M. Rybar, G. Rybkin, S. Ryu, A. Ryzhov, G. F. Rzehorz, A. F. Saavedra, G. Sabato, S. Sacerdoti, H. F.-W. Sadrozinski, R. Sadykov, F. Safai Tehrani, P. Saha, M. Sahinsoy, M. Saimpert, M. Saito, T. Saito, H. Sakamoto, Y. Sakurai, G. Salamanna, J. E. Salazar Loyola, D. Salek, P. H. Sales DeBruin, D. Salihagic, A. Salnikov, J. Salt, D. Salvatore, F. Salvatore, A. Salvucci, A. Salzburger, D. Sammel, D. Sampsonidis, D. Sampsonidou, J. Sánchez, V. Sanchez Martinez, A. Sanchez Pineda, H. Sandaker, R. L. Sandbach, C. O. Sander, M. Sandhoff, C. Sandoval, D. P. C. Sankey, M. Sannino, Y. Sano, A. Sansoni, C. Santoni, H. Santos, I. Santoyo Castillo, A. Sapronov, J. G. Saraiva, B. Sarrazin, O. Sasaki, K. Sato, E. Sauvan, G. Savage, P. Savard, N. Savic, C. Sawyer, L. Sawyer, J. Saxon, C. Sbarra, A. Sbrizzi, T. Scanlon, D. A. Scannicchio, J. Schaarschmidt, P. Schacht, B. M. Schachtner, D. Schaefer, L. Schaefer, R. Schaefer, J. Schaeffer, S. Schaepe, S. Schaetzel, U. Schäfer, A. C. Schaffer, D. Schaile, R. D. Schamberger, V. A. Schegelsky, D. Scheirich, M. Schernau, C. Schiavi, S. Schier, L. K. Schildgen, C. Schillo, M. Schioppa, S. Schlenker, K. R. Schmidt-Sommerfeld, K. Schmieden, C. Schmitt, S. Schmitt, S. Schmitz, U. Schnoor, L. Schoeffel, A. Schoening, B. D. Schoenrock, E. Schopf, M. Schott, J. F. P. Schouwenberg, J. Schovancova, S. Schramm, N. Schuh, A. Schulte, M. J. Schultens, H.-C. Schultz-Coulon, H. Schulz, M. Schumacher, B. A. Schumm, Ph. Schune, A. Schwartzman, T. A. Schwarz, H. Schweiger, Ph. Schwemling, R. Schwienhorst, J. Schwindling, A. Sciandra, G. Sciolla, M. Scornajenghi, F. Scuri, F. Scutti, J. Searcy, P. Seema, S. C. Seidel, A. Seiden, J. M. Seixas, G. Sekhniaidze, K. Sekhon, S. J. Sekula, N. Semprini-Cesari, S. Senkin, C. Serfon, L. Serin, L. Serkin, M. Sessa, R. Seuster, H. Severini, T. Sfiligoj, F. Sforza, A. Sfyrla, E. Shabalina, N. W. Shaikh, L. Y. Shan, R. Shang, J. T. Shank, M. Shapiro, P. B. Shatalov, K. Shaw, S. M. Shaw, A. Shcherbakova, C. Y. Shehu, Y. Shen, N. Sherafati, A. D. Sherman, P. Sherwood, L. Shi, S. Shimizu, C. O. Shimmin, M. Shimojima, I. P. J. Shipsey, S. Shirabe, M. Shiyakova, J. Shlomi, A. Shmeleva, D. Shoaleh Saadi, M. J. Shochet, S. Shojaii, D. R. Shope, S. Shrestha, E. Shulga, M. A. Shupe, P. Sicho, A. M. Sickles, P. E. Sidebo, E. Sideras Haddad, O. Sidiropoulou, A. Sidoti, F. Siegert, Dj. Sijacki, J. Silva, S. B. Silverstein, V. Simak, L. Simic, S. Simion, E. Simioni, B. Simmons, M. Simon, P. Sinervo, N. B. Sinev, M. Sioli, G. Siragusa, I. Siral, S. Yu. Sivoklokov, J. Sjölin, M. B. Skinner, P. Skubic, M. Slater, T. Slavicek, M. Slawinska, K. Sliwa, R. Slovak, V. Smakhtin, B. H. Smart, J. Smiesko, N. Smirnov, S. Yu. Smirnov, Y. Smirnov, L. N. Smirnova, O. Smirnova, J. W. Smith, M. N. K. Smith, R. W. Smith, M. Smizanska, K. Smolek, A. A. Snesarev, I. M. Snyder, S. Snyder, R. Sobie, F. Socher, A. Soffer, A. Søgaard, D. A. Soh, G. Sokhrannyi, C. A. Solans Sanchez, M. Solar, E. Yu. Soldatov, U. Soldevila, A. A. Solodkov, A. Soloshenko, O. V. Solovyanov, V. Solovyev, P. Sommer, H. Son, A. Sopczak, D. Sosa, C. L. Sotiropoulou, S. Sottocornola, R. Soualah, A. M. Soukharev, D. South, B. C. Sowden, S. Spagnolo, M. Spalla, M. Spangenberg, F. Spanò, D. Sperlich, F. Spettel, T. M. Spieker, R. Spighi, G. Spigo, L. A. Spiller, M. Spousta, R. D. St. Denis, A. Stabile, R. Stamen, S. Stamm, E. Stanecka, R. W. Stanek, C. Stanescu, M. M. Stanitzki, B. S. Stapf, S. Stapnes, E. A. Starchenko, G. H. Stark, J. Stark, S. H Stark, P. Staroba, P. Starovoitov, S. Stärz, R. Staszewski, M. Stegler, P. Steinberg, B. Stelzer, H. J. Stelzer, O. Stelzer-Chilton, H. Stenzel, T. J. Stevenson, G. A. Stewart, M. C. Stockton, M. Stoebe, G. Stoicea, P. Stolte, S. Stonjek, A. R. Stradling, A. Straessner, M. E. Stramaglia, J. Strandberg, S. Strandberg, M. Strauss, P. Strizenec, R. Ströhmer, D. M. Strom, R. Stroynowski, A. Strubig, S. A. Stucci, B. Stugu, N. A. Styles, D. Su, J. Su, S. Suchek, Y. Sugaya, M. Suk, V. V. Sulin, D. M. S. Sultan, S. Sultansoy, T. Sumida, S. Sun, X. Sun, K. Suruliz, C. J. E. Suster, M. R. Sutton, S. Suzuki, M. Svatos, M. Swiatlowski, S. P. Swift, I. Sykora, T. Sykora, D. Ta, K. Tackmann, J. Taenzer, A. Taffard, R. Tafirout, E. Tahirovic, N. Taiblum, H. Takai, R. Takashima, E. H. Takasugi, K. Takeda, T. Takeshita, Y. Takubo, M. Talby, A. A. Talyshev, J. Tanaka, M. Tanaka, R. Tanaka, S. Tanaka, R. Tanioka, B. B. Tannenwald, S. Tapia Araya, S. Tapprogge, S. Tarem, G. F. Tartarelli, P. Tas, M. Tasevsky, T. Tashiro, E. Tassi, A. Tavares Delgado, Y. Tayalati, A. C. Taylor, A. J. Taylor, G. N. Taylor, P. T. E. Taylor, W. Taylor, P. Teixeira-Dias, D. Temple, H. Ten Kate, P. K. Teng, J. J. Teoh, F. Tepel, S. Terada, K. Terashi, J. Terron, S. Terzo, M. Testa, R. J. Teuscher, S. J. Thais, T. Theveneaux-Pelzer, F. Thiele, J. P. Thomas, J. Thomas-Wilsker, P. D. Thompson, A. S. Thompson, L. A. Thomsen, E. Thomson, Y. Tian, M. J. Tibbetts, R. E. Ticse Torres, V. O. Tikhomirov, Yu. A. Tikhonov, S. Timoshenko, P. Tipton, S. Tisserant, K. Todome, S. Todorova-Nova, S. Todt, J. Tojo, S. Tokár, K. Tokushuku, E. Tolley, L. Tomlinson, M. Tomoto, L. Tompkins, K. Toms, B. Tong, P. Tornambe, E. Torrence, H. Torres, E. Torró Pastor, J. Toth, F. Touchard, D. R. Tovey, C. J. Treado, T. Trefzger, F. Tresoldi, A. Tricoli, I. M. Trigger, S. Trincaz-Duvoid, M. F. Tripiana, W. Trischuk, B. Trocmé, A. Trofymov, C. Troncon, M. Trottier-McDonald, M. Trovatelli, L. Truong, M. Trzebinski, A. Trzupek, K. W. Tsang, J. C.-L. Tseng, P. V. Tsiareshka, G. Tsipolitis, N. Tsirintanis, S. Tsiskaridze, V. Tsiskaridze, E. G. Tskhadadze, I. I. Tsukerman, V. Tsulaia, S. Tsuno, D. Tsybychev, Y. Tu, A. Tudorache, V. Tudorache, T. T. Tulbure, A. N. Tuna, S. Turchikhin, D. Turgeman, I. Turk Cakir, R. Turra, P. M. Tuts, G. Ucchielli, I. Ueda, M. Ughetto, F. Ukegawa, G. Unal, A. Undrus, G. Unel, F. C. Ungaro, Y. Unno, K. Uno, C. Unverdorben, J. Urban, P. Urquijo, P. Urrejola, G. Usai, J. Usui, L. Vacavant, V. Vacek, B. Vachon, K. O. H. Vadla, A. Vaidya, C. Valderanis, E. Valdes Santurio, M. Valente, S. Valentinetti, A. Valero, L. Valéry, S. Valkar, A. Vallier, J. A. Valls Ferrer, W. Van Den Wollenberg, H. van der Graaf, P. van Gemmeren, J. Van Nieuwkoop, I. van Vulpen, M. C. van Woerden, M. Vanadia, W. Vandelli, A. Vaniachine, P. Vankov, G. Vardanyan, R. Vari, E. W. Varnes, C. Varni, T. Varol, D. Varouchas, A. Vartapetian, K. E. Varvell, J. G. Vasquez, G. A. Vasquez, F. Vazeille, D. Vazquez Furelos, T. Vazquez Schroeder, J. Veatch, V. Veeraraghavan, L. M. Veloce, F. Veloso, S. Veneziano, A. Ventura, M. Venturi, N. Venturi, A. Venturini, V. Vercesi, M. Verducci, W. Verkerke, A. T. Vermeulen, J. C. Vermeulen, M. C. Vetterli, N. Viaux Maira, O. Viazlo, I. Vichou, T. Vickey, O. E. Vickey Boeriu, G. H. A. Viehhauser, S. Viel, L. Vigani, M. Villa, M. Villaplana Perez, E. Vilucchi, M. G. Vincter, V. B. Vinogradov, A. Vishwakarma, C. Vittori, I. Vivarelli, S. Vlachos, M. Vogel, P. Vokac, G. Volpi, H. von der Schmitt, E. von Toerne, V. Vorobel, K. Vorobev, M. Vos, R. Voss, J. H. Vossebeld, N. Vranjes, M. Vranjes Milosavljevic, V. Vrba, M. Vreeswijk, R. Vuillermet, I. Vukotic, P. Wagner, W. Wagner, J. Wagner-Kuhr, H. Wahlberg, S. Wahrmund, K. Wakamiya, J. Walder, R. Walker, W. Walkowiak, V. Wallangen, C. Wang, C. Wang, F. Wang, H. Wang, H. Wang, J. Wang, J. Wang, Q. Wang, R.-J. Wang, R. Wang, S. M. Wang, T. Wang, W. Wang, W. Wang, Z. Wang, C. Wanotayaroj, A. Warburton, C. P. Ward, D. R. Wardrope, A. Washbrook, P. M. Watkins, A. T. Watson, M. F. Watson, G. Watts, S. Watts, B. M. Waugh, A. F. Webb, S. Webb, M. S. Weber, S. M. Weber, S. W. Weber, S. A. Weber, J. S. Webster, A. R. Weidberg, B. Weinert, J. Weingarten, M. Weirich, C. Weiser, H. Weits, P. S. Wells, T. Wenaus, T. Wengler, S. Wenig, N. Wermes, M. D. Werner, P. Werner, M. Wessels, T. D. Weston, K. Whalen, N. L. Whallon, A. M. Wharton, A. S. White, A. White, M. J. White, R. White, D. Whiteson, B. W. Whitmore, F. J. Wickens, W. Wiedenmann, M. Wielers, C. Wiglesworth, L. A. M. Wiik-Fuchs, A. Wildauer, F. Wilk, H. G. Wilkens, H. H. Williams, S. Williams, C. Willis, S. Willocq, J. A. Wilson, I. Wingerter-Seez, E. Winkels, F. Winklmeier, O. J. Winston, B. T. Winter, M. Wittgen, M. Wobisch, A. Wolf, T. M. H. Wolf, R. Wolff, M. W. Wolter, H. Wolters, V. W. S. Wong, N. L. Woods, S. D. Worm, B. K. Wosiek, J. Wotschack, K. W. Wozniak, M. Wu, S. L. Wu, X. Wu, Y. Wu, T. R. Wyatt, B. M. Wynne, S. Xella, Z. Xi, L. Xia, D. Xu, L. Xu, T. Xu, W. Xu, B. Yabsley, S. Yacoob, D. Yamaguchi, Y. Yamaguchi, A. Yamamoto, S. Yamamoto, T. Yamanaka, F. Yamane, M. Yamatani, T. Yamazaki, Y. Yamazaki, Z. Yan, H. Yang, H. Yang, Y. Yang, Z. Yang, W.-M. Yao, Y. C. Yap, Y. Yasu, E. Yatsenko, K. H. Yau Wong, J. Ye, S. Ye, I. Yeletskikh, E. Yigitbasi, E. Yildirim, K. Yorita, K. Yoshihara, C. Young, C. J. S. Young, J. Yu, J. Yu, S. P. Y. Yuen, I. Yusuff, B. Zabinski, G. Zacharis, R. Zaidan, A. M. Zaitsev, N. Zakharchuk, J. Zalieckas, A. Zaman, S. Zambito, D. Zanzi, C. Zeitnitz, G. Zemaityte, A. Zemla, J. C. Zeng, Q. Zeng, O. Zenin, T. Ženiš, D. Zerwas, D. Zhang, D. Zhang, F. Zhang, G. Zhang, H. Zhang, J. Zhang, L. Zhang, L. Zhang, M. Zhang, P. Zhang, R. Zhang, R. Zhang, X. Zhang, Y. Zhang, Z. Zhang, X. Zhao, Y. Zhao, Z. Zhao, A. Zhemchugov, B. Zhou, C. Zhou, L. Zhou, M. Zhou, M. Zhou, N. Zhou, Y. Zhou, C. G. Zhu, H. Zhu, J. Zhu, Y. Zhu, X. Zhuang, K. Zhukov, A. Zibell, D. Zieminska, N. I. Zimine, C. Zimmermann, S. Zimmermann, Z. Zinonos, M. Zinser, M. Ziolkowski, L. Živković, G. Zobernig, A. Zoccoli, R. Zou, M. zur Nedden, L. Zwalinski, ATLAS Collaboration

**Affiliations:** 1 Department of Physics, University of Adelaide, Adelaide, Australia; 2 Physics Department, SUNY Albany, Albany, NY USA; 3 Department of Physics, University of Alberta, Edmonton, AB Canada; 4 Department of Physics, Ankara University, Ankara, Turkey; 5 Istanbul Aydin University, Istanbul, Turkey; 6 Division of Physics, TOBB University of Economics and Technology, Ankara, Turkey; 7 LAPP, CNRS/IN2P3 and Université Savoie Mont Blanc, Annecy-le-Vieux, France; 8 High Energy Physics Division, Argonne National Laboratory, Argonne, IL USA; 9 Department of Physics, University of Arizona, Tucson, AZ USA; 10 Department of Physics, The University of Texas at Arlington, Arlington, TX USA; 11 Physics Department, National and Kapodistrian University of Athens, Athens, Greece; 12 Physics Department, National Technical University of Athens, Zografou, Greece; 13 Department of Physics, The University of Texas at Austin, Austin, TX USA; 14 Institute of Physics, Azerbaijan Academy of Sciences, Baku, Azerbaijan; 15 Institut de Física d’Altes Energies (IFAE), The Barcelona Institute of Science and Technology, Barcelona, Spain; 16 Institute of Physics, University of Belgrade, Belgrade, Serbia; 17 Department for Physics and Technology, University of Bergen, Bergen, Norway; 18 Lawrence Berkeley National Laboratory, Physics Division, University of California, Berkeley, CA USA; 19 Department of Physics, Humboldt University, Berlin, Germany; 20 Laboratory for High Energy Physics, Albert Einstein Center for Fundamental Physics, University of Bern, Bern, Switzerland; 21 School of Physics and Astronomy, University of Birmingham, Birmingham, UK; 22 Department of Physics, Bogazici University, Istanbul, Turkey; 23 Department of Physics Engineering, Gaziantep University, Gaziantep, Turkey; 24 Faculty of Engineering and Natural Sciences, Istanbul Bilgi University, Istanbul, Turkey; 25 Faculty of Engineering and Natural Sciences, Bahcesehir University, Istanbul, Turkey; 26 Centro de Investigaciones, Universidad Antonio Narino, Bogota, Colombia; 27 INFN Sezione di Bologna, Bologna, Italy; 28 Dipartimento di Fisica e Astronomia, Università di Bologna, Bologna, Italy; 29 Physikalisches Institut, University of Bonn, Bonn, Germany; 30 Department of Physics, Boston University, Boston, MA USA; 31 Department of Physics, Brandeis University, Waltham, MA USA; 32 Universidade Federal do Rio De Janeiro COPPE/EE/IF, Rio de Janeiro, Brazil; 33 Electrical Circuits Department, Federal University of Juiz de Fora (UFJF), Juiz de Fora, Brazil; 34 Federal University of Sao Joao del Rei (UFSJ), Sao Joao del Rei, Brazil; 35 Instituto de Fisica, Universidade de Sao Paulo, São Paulo, Brazil; 36 Physics Department, Brookhaven National Laboratory, Upton, NY USA; 37 Transilvania University of Brasov, Brasov, Romania; 38 Horia Hulubei National Institute of Physics and Nuclear Engineering, Bucharest, Romania; 39 Department of Physics, Alexandru Ioan Cuza University of Iasi, Iasi, Romania; 40 Physics Department, National Institute for Research and Development of Isotopic and Molecular Technologies, Cluj Napoca, Romania; 41 University Politehnica Bucharest, Bucharest, Romania; 42 West University in Timisoara, Timisoara, Romania; 43 Departamento de Física, Universidad de Buenos Aires, Buenos Aires, Argentina; 44 Cavendish Laboratory, University of Cambridge, Cambridge, UK; 45 Department of Physics, Carleton University, Ottawa, ON Canada; 46 CERN, Geneva, Switzerland; 47 Enrico Fermi Institute, University of Chicago, Chicago, IL USA; 48 Departamento de Física, Pontificia Universidad Católica de Chile, Santiago, Chile; 49 Departamento de Física, Universidad Técnica Federico Santa María, Valparaiso, Chile; 50 Institute of High Energy Physics, Chinese Academy of Sciences, Beijing, China; 51 Department of Physics, Nanjing University, Nanjing, Jiangsu China; 52 Physics Department, Tsinghua University, Beijing, 100084 China; 53 University of Chinese Academy of Science (UCAS), Beijing, China; 54 Department of Modern Physics and State Key Laboratory of Particle Detection and Electronics, University of Science and Technology of China, Anhui, China; 55 School of Physics, Shandong University, Shandong, China; 56 Department of Physics and Astronomy, Key Laboratory for Particle Physics, Astrophysics and Cosmology, Ministry of Education, Shanghai Key Laboratory for Particle Physics and Cosmology, Shanghai Jiao Tong University, Shanghai (also at PKU-CHEP), Shanghai, China; 57 Université Clermont Auvergne, CNRS/IN2P3, LPC, Clermont-Ferrand, France; 58 Nevis Laboratory, Columbia University, Irvington, NY USA; 59 Niels Bohr Institute, University of Copenhagen, Kobenhavn, Denmark; 60 INFN Gruppo Collegato di Cosenza, Laboratori Nazionali di Frascati, Frascati, Italy; 61 Dipartimento di Fisica, Università della Calabria, Rende, Italy; 62 Faculty of Physics and Applied Computer Science, AGH University of Science and Technology, Kraków, Poland; 63 Marian Smoluchowski Institute of Physics, Jagiellonian University, Kraków, Poland; 64 Institute of Nuclear Physics, Polish Academy of Sciences, Kraków, Poland; 65 Physics Department, Southern Methodist University, Dallas, TX USA; 66 Physics Department, University of Texas at Dallas, Richardson, TX USA; 67 DESY, Hamburg and Zeuthen, Germany; 68 Lehrstuhl für Experimentelle Physik IV, Technische Universität Dortmund, Dortmund, Germany; 69 Institut für Kern- und Teilchenphysik, Technische Universität Dresden, Dresden, Germany; 70 Department of Physics, Duke University, Durham, NC USA; 71 SUPA-School of Physics and Astronomy, University of Edinburgh, Edinburgh, UK; 72 INFN e Laboratori Nazionali di Frascati, Frascati, Italy; 73 Fakultät für Mathematik und Physik, Albert-Ludwigs-Universität, Freiburg, Germany; 74 Departement de Physique Nucleaire et Corpusculaire, Université de Genève, Geneva, Switzerland; 75 INFN Sezione di Genova, Genoa, Italy; 76 Dipartimento di Fisica, Università di Genova, Genoa, Italy; 77 E. Andronikashvili Institute of Physics, Iv. Javakhishvili Tbilisi State University, Tbilisi, Georgia; 78 High Energy Physics Institute, Tbilisi State University, Tbilisi, Georgia; 79 II Physikalisches Institut, Justus-Liebig-Universität Giessen, Giessen, Germany; 80 SUPA-School of Physics and Astronomy, University of Glasgow, Glasgow, UK; 81 II Physikalisches Institut, Georg-August-Universität, Göttingen, Germany; 82 Laboratoire de Physique Subatomique et de Cosmologie, Université Grenoble-Alpes, CNRS/IN2P3, Grenoble, France; 83 Laboratory for Particle Physics and Cosmology, Harvard University, Cambridge, MA USA; 84 Kirchhoff-Institut für Physik, Ruprecht-Karls-Universität Heidelberg, Heidelberg, Germany; 85 Physikalisches Institut, Ruprecht-Karls-Universität Heidelberg, Heidelberg, Germany; 86 Faculty of Applied Information Science, Hiroshima Institute of Technology, Hiroshima, Japan; 87 Department of Physics, The Chinese University of Hong Kong, Shatin, N.T. Hong Kong; 88 Department of Physics, The University of Hong Kong, Hong Kong, China; 89 Department of Physics, Institute for Advanced Study, The Hong Kong University of Science and Technology, Clear Water Bay, Kowloon, Hong Kong, China; 90 Department of Physics, National Tsing Hua University, Taiwan, Taiwan; 91 Department of Physics, Indiana University, Bloomington, IN USA; 92 Institut für Astro- und Teilchenphysik, Leopold-Franzens-Universität, Innsbruck, Austria; 93 University of Iowa, Iowa City, IA USA; 94 Department of Physics and Astronomy, Iowa State University, Ames, IA USA; 95 Joint Institute for Nuclear Research, JINR Dubna, Dubna, Russia; 96 KEK, High Energy Accelerator Research Organization, Tsukuba, Japan; 97 Graduate School of Science, Kobe University, Kobe, Japan; 98 Faculty of Science, Kyoto University, Kyoto, Japan; 99 Kyoto University of Education, Kyoto, Japan; 100 Research Center for Advanced Particle Physics and Department of Physics, Kyushu University, Fukuoka, Japan; 101 Instituto de Física La Plata, Universidad Nacional de La Plata and CONICET, La Plata, Argentina; 102 Physics Department, Lancaster University, Lancaster, UK; 103 INFN Sezione di Lecce, Lecce, Italy; 104 Dipartimento di Matematica e Fisica, Università del Salento, Lecce, Italy; 105 Oliver Lodge Laboratory, University of Liverpool, Liverpool, UK; 106 Department of Experimental Particle Physics, Jožef Stefan Institute and Department of Physics, University of Ljubljana, Ljubljana, Slovenia; 107 School of Physics and Astronomy, Queen Mary University of London, London, UK; 108 Department of Physics, Royal Holloway University of London, Surrey, UK; 109 Department of Physics and Astronomy, University College London, London, UK; 110 Louisiana Tech University, Ruston, LA USA; 111 Laboratoire de Physique Nucléaire et de Hautes Energies, UPMC and Université Paris-Diderot and CNRS/IN2P3, Paris, France; 112 Fysiska institutionen, Lunds universitet, Lund, Sweden; 113 Departamento de Fisica Teorica C-15, Universidad Autonoma de Madrid, Madrid, Spain; 114 Institut für Physik, Universität Mainz, Mainz, Germany; 115 School of Physics and Astronomy, University of Manchester, Manchester, UK; 116 CPPM, Aix-Marseille Université and CNRS/IN2P3, Marseille, France; 117 Department of Physics, University of Massachusetts, Amherst, MA USA; 118 Department of Physics, McGill University, Montreal, QC Canada; 119 School of Physics, University of Melbourne, Victoria, Australia; 120 Department of Physics, The University of Michigan, Ann Arbor, MI USA; 121 Department of Physics and Astronomy, Michigan State University, East Lansing, MI USA; 122 INFN Sezione di Milano, Milan, Italy; 123 Dipartimento di Fisica, Università di Milano, Milan, Italy; 124 B.I. Stepanov Institute of Physics, National Academy of Sciences of Belarus, Minsk, Republic of Belarus; 125 Research Institute for Nuclear Problems of Byelorussian State University, Minsk, Republic of Belarus; 126 Group of Particle Physics, University of Montreal, Montreal, QC Canada; 127 P.N. Lebedev Physical Institute of the Russian Academy of Sciences, Moscow, Russia; 128 Institute for Theoretical and Experimental Physics (ITEP), Moscow, Russia; 129 National Research Nuclear University MEPhI, Moscow, Russia; 130 D.V. Skobeltsyn Institute of Nuclear Physics, M.V. Lomonosov Moscow State University, Moscow, Russia; 131 Fakultät für Physik, Ludwig-Maximilians-Universität München, Munich, Germany; 132 Max-Planck-Institut für Physik (Werner-Heisenberg-Institut), Munich, Germany; 133 Nagasaki Institute of Applied Science, Nagasaki, Japan; 134 Graduate School of Science and Kobayashi-Maskawa Institute, Nagoya University, Nagoya, Japan; 135 INFN Sezione di Napoli, Naples, Italy; 136 Dipartimento di Fisica, Università di Napoli, Naples, Italy; 137 Department of Physics and Astronomy, University of New Mexico, Albuquerque, NM USA; 138 Institute for Mathematics, Astrophysics and Particle Physics, Radboud University Nijmegen/Nikhef, Nijmegen, The Netherlands; 139 Nikhef National Institute for Subatomic Physics, University of Amsterdam, Amsterdam, The Netherlands; 140 Department of Physics, Northern Illinois University, DeKalb, IL USA; 141 Budker Institute of Nuclear Physics, SB RAS, Novosibirsk, Russia; 142 Department of Physics, New York University, New York, NY USA; 143 Ohio State University, Columbus, OH USA; 144 Faculty of Science, Okayama University, Okayama, Japan; 145 Homer L. Dodge Department of Physics and Astronomy, University of Oklahoma, Norman, OK USA; 146 Department of Physics, Oklahoma State University, Stillwater, OK USA; 147 Palacký University, RCPTM, Olomouc, Czech Republic; 148 Center for High Energy Physics, University of Oregon, Eugene, OR USA; 149 LAL, Univ. Paris-Sud, CNRS/IN2P3, Université Paris-Saclay, Orsay, France; 150 Graduate School of Science, Osaka University, Osaka, Japan; 151 Department of Physics, University of Oslo, Oslo, Norway; 152 Department of Physics, Oxford University, Oxford, UK; 153 INFN Sezione di Pavia, Pavia, Italy; 154 Dipartimento di Fisica, Università di Pavia, Pavia, Italy; 155 Department of Physics, University of Pennsylvania, Philadelphia, PA USA; 156 National Research Centre “Kurchatov Institute” B.P. Konstantinov Petersburg Nuclear Physics Institute, St. Petersburg, Russia; 157 INFN Sezione di Pisa, Pisa, Italy; 158 Dipartimento di Fisica E. Fermi, Università di Pisa, Pisa, Italy; 159 Department of Physics and Astronomy, University of Pittsburgh, Pittsburgh, PA USA; 160 Laboratório de Instrumentação e Física Experimental de Partículas-LIP, Lisboa, Portugal; 161 Faculdade de Ciências, Universidade de Lisboa, Lisbon, Portugal; 162 Department of Physics, University of Coimbra, Coimbra, Portugal; 163 Centro de Física Nuclear da Universidade de Lisboa, Lisbon, Portugal; 164 Departamento de Fisica, Universidade do Minho, Braga, Portugal; 165 Departamento de Fisica Teorica y del Cosmos, Universidad de Granada, Granada, Spain; 166 Dep Fisica and CEFITEC of Faculdade de Ciencias e Tecnologia, Universidade Nova de Lisboa, Caparica, Portugal; 167 Institute of Physics, Academy of Sciences of the Czech Republic, Prague, Czech Republic; 168 Czech Technical University in Prague, Prague, Czech Republic; 169 Faculty of Mathematics and Physics, Charles University, Prague, Czech Republic; 170 State Research Center Institute for High Energy Physics (Protvino), NRC KI, Protvino, Russia; 171 Particle Physics Department, Rutherford Appleton Laboratory, Didcot, UK; 172 INFN Sezione di Roma, Rome, Italy; 173 Dipartimento di Fisica, Sapienza Università di Roma, Rome, Italy; 174 INFN Sezione di Roma Tor Vergata, Rome, Italy; 175 Dipartimento di Fisica, Università di Roma Tor Vergata, Rome, Italy; 176 INFN Sezione di Roma Tre, Rome, Italy; 177 Dipartimento di Matematica e Fisica, Università Roma Tre, Rome, Italy; 178 Faculté des Sciences Ain Chock, Réseau Universitaire de Physique des Hautes Energies-Université Hassan II, Casablanca, Morocco; 179 Centre National de l’Energie des Sciences Techniques Nucleaires, Rabat, Morocco; 180 Faculté des Sciences Semlalia, Université Cadi Ayyad, LPHEA-Marrakech, Marrakech, Morocco; 181 Faculté des Sciences, Université Mohamed Premier and LPTPM, Oujda, Morocco; 182 Faculté des Sciences, Université Mohammed V, Rabat, Morocco; 183 DSM/IRFU (Institut de Recherches sur les Lois Fondamentales de l’Univers), CEA Saclay (Commissariat à l’Energie Atomique et aux Energies Alternatives), Gif-sur-Yvette, France; 184 Santa Cruz Institute for Particle Physics, University of California Santa Cruz, Santa Cruz, CA USA; 185 Department of Physics, University of Washington, Seattle, WA USA; 186 Department of Physics and Astronomy, University of Sheffield, Sheffield, UK; 187 Department of Physics, Shinshu University, Nagano, Japan; 188 Department Physik, Universität Siegen, Siegen, Germany; 189 Department of Physics, Simon Fraser University, Burnaby, BC Canada; 190 SLAC National Accelerator Laboratory, Stanford, CA USA; 191 Faculty of Mathematics, Physics and Informatics, Comenius University, Bratislava, Slovak Republic; 192 Department of Subnuclear Physics, Institute of Experimental Physics of the Slovak Academy of Sciences, Kosice, Slovak Republic; 193 Department of Physics, University of Cape Town, Cape Town, South Africa; 194 Department of Physics, University of Johannesburg, Johannesburg, South Africa; 195 School of Physics, University of the Witwatersrand, Johannesburg, South Africa; 196 Department of Physics, Stockholm University, Stockholm, Sweden; 197 The Oskar Klein Centre, Stockholm, Sweden; 198 Physics Department, Royal Institute of Technology, Stockholm, Sweden; 199 Departments of Physics and Astronomy and Chemistry, Stony Brook University, Stony Brook, NY USA; 200 Department of Physics and Astronomy, University of Sussex, Brighton, UK; 201 School of Physics, University of Sydney, Sydney, Australia; 202 Institute of Physics, Academia Sinica, Taipei, Taiwan; 203 Department of Physics, Technion: Israel Institute of Technology, Haifa, Israel; 204 Raymond and Beverly Sackler School of Physics and Astronomy, Tel Aviv University, Tel Aviv, Israel; 205 Department of Physics, Aristotle University of Thessaloniki, Thessaloniki, Greece; 206 International Center for Elementary Particle Physics and Department of Physics, The University of Tokyo, Tokyo, Japan; 207 Graduate School of Science and Technology, Tokyo Metropolitan University, Tokyo, Japan; 208 Department of Physics, Tokyo Institute of Technology, Tokyo, Japan; 209 Tomsk State University, Tomsk, Russia; 210 Department of Physics, University of Toronto, Toronto, ON Canada; 211 INFN-TIFPA, Trento, Italy; 212 University of Trento, Trento, Italy; 213 TRIUMF, Vancouver, BC Canada; 214 Department of Physics and Astronomy, York University, Toronto, ON Canada; 215 Faculty of Pure and Applied Sciences, and Center for Integrated Research in Fundamental Science and Engineering, University of Tsukuba, Tsukuba, Japan; 216 Department of Physics and Astronomy, Tufts University, Medford, MA USA; 217 Department of Physics and Astronomy, University of California Irvine, Irvine, CA USA; 218 INFN Gruppo Collegato di Udine, Sezione di Trieste, Udine, Italy; 219 ICTP, Trieste, Italy; 220 Dipartimento di Chimica, Fisica e Ambiente, Università di Udine, Udine, Italy; 221 Department of Physics and Astronomy, University of Uppsala, Uppsala, Sweden; 222 Department of Physics, University of Illinois, Urbana, IL USA; 223 Instituto de Fisica Corpuscular (IFIC), Centro Mixto Universidad de Valencia, CSIC, Valencia, Spain; 224 Department of Physics, University of British Columbia, Vancouver, BC Canada; 225 Department of Physics and Astronomy, University of Victoria, Victoria, BC Canada; 226 Department of Physics, University of Warwick, Coventry, UK; 227 Waseda University, Tokyo, Japan; 228 Department of Particle Physics, The Weizmann Institute of Science, Rehovot, Israel; 229 Department of Physics, University of Wisconsin, Madison, WI USA; 230 Fakultät für Physik und Astronomie, Julius-Maximilians-Universität, Würzburg, Germany; 231 Fakultät für Mathematik und Naturwissenschaften, Fachgruppe Physik, Bergische Universität Wuppertal, Wuppertal, Germany; 232 Department of Physics, Yale University, New Haven, CT USA; 233 Yerevan Physics Institute, Yerevan, Armenia; 234 Centre de Calcul de l’Institut National de Physique Nucléaire et de Physique des Particules (IN2P3), Villeurbanne, France; 235 Academia Sinica Grid Computing, Institute of Physics, Academia Sinica, Taipei, Taiwan; 236 CERN, 1211 Geneva 23, Switzerland

## Abstract

This paper presents a direct measurement of the decay width of the top quark using $t\bar{t}$ events in the lepton+jets final state. The data sample was collected by the ATLAS detector at the LHC in proton–proton collisions at a centre-of-mass energy of 8 TeV and corresponds to an integrated luminosity of 20.2 fb$^{-1}$. The decay width of the top quark is measured using a template fit to distributions of kinematic observables associated with the hadronically and semileptonically decaying top quarks. The result, $\Gamma _t = 1.76 \pm 0.33~(\text {stat.})~ ^{+0.79}_{-0.68}~(\text {syst.})~\text {GeV}$ for a top-quark mass of 172.5 GeV, is consistent with the prediction of the Standard Model.

## Introduction

The top quark is the heaviest particle in the Standard Model (SM) of elementary particle physics, discovered more than 20 years ago in 1995 [1, 2]. Due to its large mass of around 173 GeV [3–5], the lifetime of the top quark is extremely short. Hence, its decay width is the largest of all SM fermions. A next-to-leading-order (NLO) calculation predicts a decay width of $\Gamma _t = 1.33$ GeV for a top-quark mass ($m_t$) of 172.5 GeV [6, 7]. Variations of the parameters entering the NLO calculation, the *W*-boson mass, the strong coupling constant $\alpha _\text {S}$, the Fermi coupling constant $G_\text {F}$ and the Cabibbo–Kobayashi–Maskawa (CKM) matrix element $V_{tb}$, within experimental uncertainties [8] yield an uncertainty of 6%. The recent next-to-next-to-leading-order (NNLO) calculation predicts $\Gamma _t = 1.322$ GeV for $m_t=172.5$ GeV and $\alpha _\text {S} = 0.1181$ [9].

A deviation from the SM prediction could indicate non-SM decay channels of the top quark or non-SM top-quark couplings, as predicted by many beyond-the-Standard-Model (BSM) theories. The top-quark decay width can be modified by direct top-quark decays into e.g. a charged Higgs boson [10, 11] or via flavour-changing neutral currents [12, 13] or by non-SM radiative corrections [14]. Furthermore, some vector-like quark models [15] modify the $|V_{tb}|$ CKM matrix element and thus $\Gamma _t$. Precise measurements of $\Gamma _t$ can consequently restrict the parameter space of many BSM models.

Extractions of $\Gamma _t$ from the $t \rightarrow Wb$ branching ratio $\mathcal {B}$ and the single-top *t*-channel cross-section, such as those of Refs. [16, 17], have reached a precision of 0.14 GeV, but depend on the assumption that $\sum _q \mathcal {B} (t\rightarrow Wq) = 1$ with $q = d,s,b$, and use theoretical SM predictions for $\Gamma (t \rightarrow Wb)$ and the single-top *t*-channel cross-section. Some BSM models, vector-like quark models for example [15], predict a sizeable deviation from the assumptions used in indirect measurements. This provides a motivation to perform a direct measurement of $\Gamma _t$. However, such a measurement is not yet sensitive to alternative BSM models with the current precision. A direct measurement of $\Gamma _t$, based on the analysis of the top-quark invariant mass distribution was performed at the Tevatron by the CDF Collaboration [18]. A bound on the decay width of $1.10< \Gamma _t < 4.05$ GeV for $m_t = 172.5$ GeV was set at 68% confidence level. Direct measurements are limited by the experimental resolution of the top-quark mass spectrum, and so far are significantly less precise than indirect measurements, but avoid model-dependent assumptions.

This analysis is based on ATLAS data recorded at a centre-of-mass energy of $\sqrt{s} = 8$ TeV in 2012 in LHC proton–proton collisions. The top-quark decay width is extracted using $t\bar{t}$ events in the lepton+jets channel with $t\rightarrow Wb$, where one *W* boson from the two top quarks decays hadronically into a pair of quarks and the other one decays leptonically into a charged lepton and a neutrino (the corresponding top quarks are referred to as hadronically and semileptonically decaying, respectively). Thus, the final state consists of four jets, two of which are *b*-jets, one charged electron or muon and missing transverse momentum ($E_{\text {T}}^{\text {miss}}$) due to the undetected neutrino. Additional jets can originate from initial- or final-state radiation (ISR or FSR). Selected events include *W*-boson decays into a $\tau$ lepton if the $\tau$ decays leptonically.

The measurement is performed using two observables sensitive to $\Gamma _t$: $m_{\ell b}$, which is the reconstructed invariant mass of the system formed by the *b*-jet and the charged lepton $\ell$ from the semileptonic top-quark decay, and $\Delta R_{\text {min}}(j_b, j_l)$, defined as the angular distance^1^ between the *b*-jet $j_b$ associated with the hadronic top-quark decay and the closest light jet $j_l$ from the hadronically decaying *W* boson. This approach exploits the kinematic information from both the hadronically and semileptonically decaying top quarks. A template method is used to measure the top-quark decay width. Templates for the two observables are built for all contributing SM processes. Distributions for multijet production are formed using a data-driven method. Templates for the other SM processes, including top-quark pair production and electroweak single-top production, are generated using Monte Carlo (MC) simulations. Templates for different top-quark decay width values are constructed by reweighting MC events. These templates are used in a binned likelihood fit to data to extract $\Gamma _t$.

The ATLAS detector is described in the next section. Section 3 introduces MC simulated samples and the dataset, followed by a description of the event selection and reconstruction in Sect. 4. The template fit is described in Sect. 5, the systematic uncertainties are estimated in Sect. 6. Section 7 presents the results of the measurement and Sect. 8 gives the conclusions.

## ATLAS detector

The ATLAS experiment [19] at the LHC is a multi-purpose particle detector with a forward-backward symmetric cylindrical geometry and a near $4\pi$ coverage in solid angle. It consists of an inner tracking detector surrounded by a thin superconducting solenoid providing a 2 T axial magnetic field, electromagnetic and hadron calorimeters, and a muon spectrometer. The inner tracking detector covers the pseudorapidity range $|\eta | < 2.5$. It consists of silicon pixel, silicon microstrip, and transition radiation tracking detectors. Lead/liquid-argon (LAr) sampling calorimeters provide electromagnetic (EM) energy measurements with high granularity. A hadron (steel/scintillator-tile) calorimeter covers the central pseudorapidity range ($|\eta | < 1.7$). The endcap and forward regions are instrumented with LAr calorimeters for both the EM and hadronic energy measurements up to $|\eta | = 4.9$. The muon spectrometer surrounds the calorimeters and features three large air-core toroid superconducting magnets with eight coils each. The field integral of the toroids ranges between 2.0 and 6.0 Tm across most of the detector. It includes a system of precision tracking chambers and fast detectors for triggering. A three-level trigger system is used to select events. The first-level trigger is implemented in hardware and uses a subset of the detector information to reduce the accepted rate to at most 75 kHz. This is followed by two software-based trigger levels that together reduce the accepted event rate to 400 Hz on average.

## Data and simulated event samples

The decay width of the top quark is measured using data which correspond to an integrated luminosity of $20.2~\hbox {fb}^{-1}$ [20]. Single-lepton triggers for electrons and muons under stable beam conditions were used. For each lepton type, two single-lepton triggers with the transverse momentum, $p_{\mathrm{T}}$, thresholds of 24 (24) and 60 (36) GeV for electrons (muons) were used. The two triggers with the lower $p_{\mathrm{T}}$ thresholds imposed additional isolation requirements on the lepton to keep the trigger rate low. No isolation requirement was used by the higher $p_{\mathrm{T}}$ threshold triggers.

The nominal signal $t\bar{t}$ MC sample was generated assuming a top-quark mass of $m_t = 172.5$ GeV using the Powheg-Box (v1) event generator [21–23], referred to in the following as Powheg, providing NLO QCD matrix-element (ME) calculations [24]. The $h_{\text {damp}}$ parameter that regulates the high-$p_{\text {T}}$ radiation in Powheg was set to $m_t$. The CT10 parton distribution function (PDF) set [25] was used. The event generator was interfaced with Pythia 6.425 [26] for parton showering (PS), hadronisation and underlying event modelling, using the Perugia 2011C set of tuned parameters [27] and the CTEQ6L1 PDF set [28]. To estimate the impact of the parton shower and hadronisation model choice, a Powheg +Pythia 6 sample is compared to a sample generated with Powheg interfaced with Herwig 6.520 [29] using Jimmy v4.31 [30] to simulate the underlying event. The latter sample is referred to as Powheg +Herwig in the following. The $h_{\text {damp}}$ parameter was set to infinity in both samples used to assess the systematic uncertainty due to parton shower modelling. The uncertainty due to the MC event generator choice is estimated using the alternative MC event generator MC@NLO  [31, 32] for the hard process, interfaced to Herwig for showering, hadronisation and the simulation of the underlying event which is compared to the Powheg +Herwig sample. To assess the impact of the initial- and final-state radiation, samples generated with Powheg were interfaced to Pythia with different settings for the event generator parameters regulating ISR and FSR. In these samples, the $h_{\text {damp}}$ parameter and the factorisation and renormalisation scales in Powheg, as well as the transverse momentum scale for space-like parton shower evolution in Pythia were varied to cover the range in additional jet multiplicity corresponding to the uncertainty of $t\bar{t}$ production measurements in association with jets [33, 34]. The $t\bar{t}$  samples are normalised using the theoretical cross-section of $\sigma _{t\bar{t}} = 253^{+15}_{-16}$ pb, based on a calculation performed with the Top++2.0 [35–40] program that includes NNLO corrections and resums next-to-next-to-leading-logarithmic-order (NNLL) soft gluon terms. PDF and scale variations, the choice of $\alpha _\text {S}$, and the input top-quark mass are regarded as sources of systematic uncertainty.

Background events containing a *W* or a *Z* boson produced in association with jets were generated using the Alpgen 2.14 [41] LO event generator with up to five additional partons and the CTEQ6L1 PDF set [28]. Parton shower and hadronisation were modelled with Pythia 6.425. Separate samples were generated for $W/Z+b\bar{b}$, $W/Z+c\bar{c}$, $W+c$, and *W* / *Z*+light jets. A parton–jet matching scheme (“MLM matching”) [42] is used to prevent double-counting of jets generated by both the matrix-element calculation and the parton-shower evolution. The *W*+jets events are normalised using a data-driven method exploiting the asymmetry of $W^{\pm }$ production in *pp* collision [43]. The corrections for event generator mismodelling in the fractions of different flavour components ($W+b\bar{b}$, $W+c\bar{c}$, $W+c$ and *W*+light jets) are estimated in a sample with the same lepton and $E_{\text {T}}^{\text {miss}}$ selections as the signal selection, but with only two jets and no *b*-tagging requirement. The *b*-jet multiplicity, in conjunction with knowledge of the *b*-tagging and mistag efficiency, is used to extract the heavy-flavour fractions. The correction factors extracted from the MC simulation and used in the analysis are $K_{b\bar{b}} = K_{c\bar{c}} = 1.50 \pm 0.11$ (stat.+syst.), $K_c = 1.07 \pm 0.27$ (stat.+syst.) and $K_{\text {light}} = 0.80 \pm 0.04$ (stat.+syst.) [43]. The *Z*+jets events are normalised using the inclusive NNLO theoretical cross-section [44].

Diboson background samples were generated with the Sherpa 1.4.1 event generator [45] with up to three additional partons in the LO matrix elements using the CT10 PDF set. The samples are normalised with the NLO theoretical cross-sections [46].

At leading order, three single-top-quark production mechanisms, *s*-channel, *t*-channel and associated *Wt* production, contribute to the single-top-quark background. These processes were simulated with Powheg  [47, 48] using the CT10 PDF set. All samples were interfaced to Pythia 6.425 with the CTEQ6L1 PDF set and the Perugia 2011C tune. Overlaps between the $t\bar{t}$ and *Wt* final states were removed [49]. All individual single-top-quark samples are normalised using their corresponding approximate NNLO theoretical cross-sections [50, 51] based on an MSTW 2008 NNLO PDF set calculation [52].

Multijet events can pass the selection because of the misidentification of a jet or a photon as an electron or muon (fake lepton) or because of the presence of a non-prompt lepton (electron or muon), which can originate from semileptonic decays of heavy-flavour hadrons. This background, referred to as multijet background in the following, is estimated directly from data using a data-driven matrix method [53].

The detector response [54] was simulated using the GEANT 4 simulation toolkit [55]. To estimate some systematic effects, samples passed through a fast simulation [56] are used. This simulation utilises a parameterisation of the response of the EM and hadronic calorimeters while a full simulation is used for the tracking systems. The effects of in-time and out-of-time pile-up (multiple *pp* interactions from the same or neighbouring bunch-crossings) are included in these simulations. Events from minimum-bias interactions were simulated with the Pythia 8.1 event generator with the MSTW 2008 LO PDF set and the A2 tune [57] and overlaid on signal and background processes to simulate the effect of pile-up. The simulated events are reweighted in order to match the distribution of the average number of collisions per bunch crossing in the data. MC events are processed through the same reconstruction algorithms as the data.

## Event reconstruction and selection

### Event reconstruction

Electrons, muons, jets, *b*-jets and missing transverse momentum are used to select $t\bar{t}$ events in this analysis.

Electron candidates are reconstructed using energy deposits in the electromagnetic calorimeter matched to reconstructed inner-detector tracks [58]. These electron candidates are required to have $p_{\text {T}} > 25$ GeV and $|\eta | < 2.47$, with the transition region between the barrel and endcap detector $1.37< |\eta | < 1.52$ excluded. Isolation requirements are used to reduce the background from fake and non-prompt electrons. A $p_{\text {T}}$- and $\eta$-dependent isolation requirement is placed on the sum of transverse energy deposited within a cone of size $\Delta R = 0.2$ around the calorimeter cells associated to the electron. This energy sum excludes cells in the cluster associated with the electron and is corrected for leakage from that cluster and for energy deposits from pile-up. Another $p_{\text {T}}$- and $\eta$-dependent isolation requirement is made on the scalar sum of track transverse momenta around the electron within a cone of size $\Delta R = 0.3$. Furthermore, the longitudinal impact parameter $|z_0|$ of the electron track with respect to the selected event primary vertex^2^ (PV) is required to be smaller than 2 mm.

Muon candidate reconstruction is based on tracks in the muon spectrometer which are matched to inner-detector tracks [59]. The combined muon track must satisfy $p_{\text {T}} > 25$ GeV and $|\eta |<2.5$ and its longitudinal impact parameter $z_0$ with respect to the PV is required to be smaller than 2 mm. Muon candidates have to be separated from any jet by $\Delta R > 0.4$ and are required to satisfy a $p_{\text {T}}$-dependent track-based isolation requirement. Specifically, the scalar sum of the transverse momenta of tracks within a cone of size $\Delta R = 10$ GeV$/p_{\text {T}}$ around the muon candidate (excluding the muon track itself) has to be less than 5% of the muon transverse momentum.

Jets are reconstructed using the anti-$k_t$ algorithm [60], implemented in the FastJet package [61], with a radius parameter of $R=0.4$. The jet reconstruction starts from calibrated topological clusters [62] which are built from energy deposits in the calorimeters. To correct for effects due to the non-compensating calorimeter response, dead material and out-of-cluster leakage, a local cluster calibration scheme [63, 64] is applied prior to jet finding. Simulations of charged and neutral particles are exploited to estimate these corrections. The jets are calibrated by applying energy- and $|\eta |$-dependent calibration factors, derived from simulations, to the mean energy of the jets built from the stable particles [65]. In addition, a residual calibration [66] of the jet energy scale (JES) was performed using data taken in 2012. Dijet events are used to calibrate jets in the forward region against jets in the central region. Photon+jet as well as *Z*+jet events are used to calibrate central jets, and multijet events are used to calibrate high-$p_{\text {T}}$ jets. These measurements are then combined. Jets are accepted if they fulfil $p_{\text {T}} > 25$ GeV and $|\eta | < 2.5$ after energy calibration. To reduce the contribution from jets associated with pile-up, jets having $p_{\text {T}} <50$ GeV and $|\eta |<2.4$ must satisfy a requirement [67] for the jet vertex fraction,^3^ $\text {JVF} > 0.5$. To prevent double-counting of electrons as jets, the closest jet lying $\Delta R < 0.2$ of a selected electron is discarded. If the nearest jet surviving the selection described above is within $\Delta R = 0.4$ of the electron, the electron is discarded.

The purity of the selected sample is improved by tagging jets containing *b*-hadrons on the basis of their large mass and decay time. The MV1 algorithm [69] based on multivariate techniques is utilised to identify jets originating from the hadronisation of a *b*-quark. The chosen working point corresponds to an efficiency of 70% to correctly identify a *b*-quark jet in simulated $t\bar{t}$ events, with a light-jet rejection factor of around 130 and a *c*-jet rejection factor of 5. The tagging efficiencies in simulation are corrected to match the results of the calibrations based on data [70, 71].

The $E_{\text {T}}^{\text {miss}}$ serves as a measure of the transverse momentum of the neutrino which originates from the leptonically decaying *W* boson. It is calculated using all reconstructed and calibrated particles (electrons, muons, photons) and jets in the transverse plane. Contributions from unassociated energy depositions are also taken into account [72].

### Event selection

According to the signature of the $t\bar{t}$ signal in the lepton+jets decay channel, events are required to have exactly one reconstructed electron or muon and at least four jets, at least one of which is required to be *b*-tagged. This selection includes *W*-boson decays into a $\tau$ lepton if the $\tau$ decays leptonically. Events are required to pass a single-electron or single-muon trigger. If at least one of the jets having $p_{\text {T}} > 20$ GeV is identified as out-of-time activity from a previous *pp* collision, as calorimeter noise or non-collision background, the event is not considered [73].

**Fig. 1:**
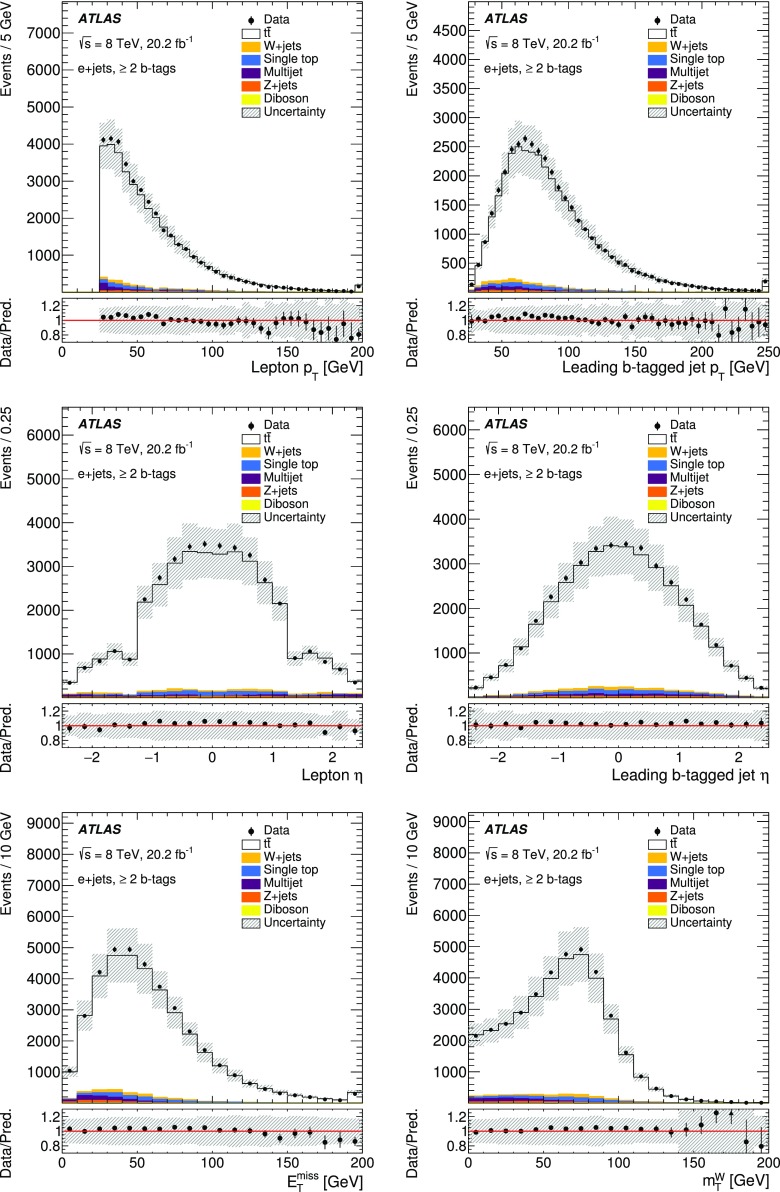
Distributions of the lepton and leading *b*-tagged jet $p_{\text {T}}$, lepton and leading *b*-tagged jet $\eta$, $E_{\text {T}}^{\text {miss}}$ and $m^W_{\text {T}}$ in the electron+jets channel for events with at least two *b*-tagged jets after event selection. The hatched bands show the normalisation uncertainty in the signal and background contributions and the signal model systematic uncertainties. The first and last bins contain underflow and overflow events, respectively

**Fig. 2:**
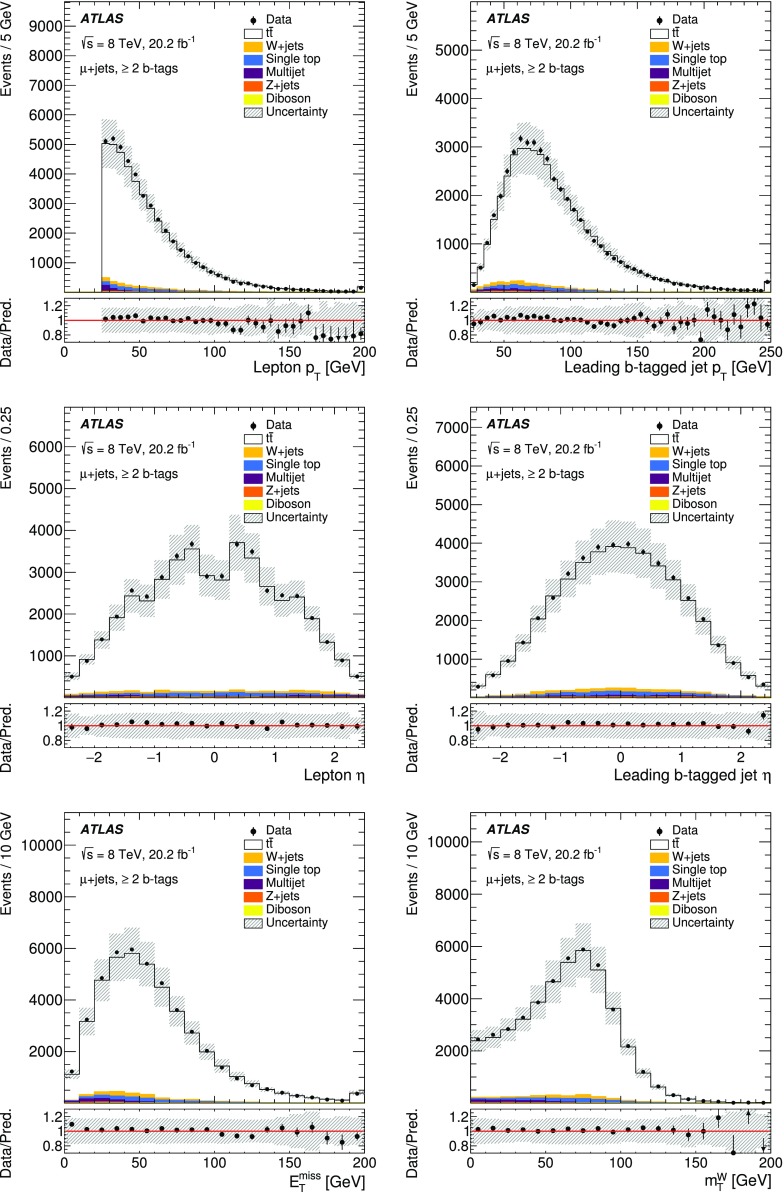
Distributions of the lepton and leading *b*-tagged jet $p_{\text {T}}$, lepton and leading *b*-tagged jet $\eta$, $E_{\text {T}}^{\text {miss}}$ and $m^W_{\text {T}}$ in the muon+jets channel for events with at least two *b*-tagged jets after event selection. The hatched bands show the normalisation uncertainty in the signal and background contributions and the signal model systematic uncertainties. The first and last bins contain underflow and overflow events, respectively

Events with exactly one *b*-tagged jet need to have $E_{\text {T}}^{\text {miss}}$ > 20 GeV and $E_{\text {T}}^{\text {miss}} + m^W_{\text {T}}> 60$ GeV, where $m^W_{\text {T}}$ is the transverse mass of the leptonically decaying *W* boson defined as $m^W_{\text {T}} = \sqrt{ 2p^{\ell }_{\text {T}} E_{\text {T}}^{\text {miss}} (1-\cos \Delta \phi (\ell ,E_{\text {T}}^{\text {miss}})) }$. These requirements suppress the background due to misidentified leptons. As this background becomes very small in high *b*-tag multiplicity regions, these requirements are not applied to events with at least two *b*-tagged jets. Selected events are reconstructed under the $t\bar{t}$ decay hypothesis using a likelihood-based method described in Sect. 4.3. The logarithm of the likelihood has to satisfy $\ln (L)> -50$ to suppress the combinatorial background due to wrongly reconstructed events and to decrease other backgrounds. This improves the sensitivity of the measurement by increasing the fraction of well-reconstructed $t\bar{t}$ events in the selected sample. Events satisfying all selection criteria are separated into eight mutually exclusive analysis regions. The events are categorised according to the flavour of the selected lepton and whether they have exactly one or at least two *b*-tagged jets. They are further split into two $|\eta |$ regions, a central region with all four jets associated with the $t\bar{t}$ decay having $|\eta | \le 1$ and a second one with at least one jet with $|\eta | > 1$. This approach takes advantage of the different sensitivity of these regions to detector resolution effects and pile-up contributions and different amounts of background. The corresponding event yields are listed in Table 1. Figures 1, 2 show the distributions of the lepton and leading *b*-tagged jet $p_{\text {T}}$, lepton and leading *b*-tagged jet $\eta$, $E_{\text {T}}^{\text {miss}}$ and $m^W_{\text {T}}$ for events with at least two *b*-tagged jets in the electron and muon channels, respectively. Good agreement within the assigned statistical and systematic uncertainties is observed between data and the predictions from simulation.

**Table 1:** Event yields after the event selection in the (a) electron+jets and (b) muon+jets channel for events with exactly one or at least two *b*-tags divided into events where all four jets associated with the $t\bar{t}$ decay have $|\eta | \le 1$ and events where at least one jet has $|\eta | > 1$. The uncertainties in the signal and background yields arising from normalisation uncertainties of each sample are shown. These correspond to the theory uncertainties as described in Sect. 5 for the background sources except for the *W*+jets and the multijet background, whose uncertainties originate from the data-driven methods

Sample	$|\eta | \le 1$ region		$|\eta | > 1$ region	
	1 *b*-tag	$\ge 2$ *b*-tags	1 *b*-tag	$\ge 2$ *b*-tags
(a) Electron+jets (*e*+jets) channel				
$t\bar{t}$	$5850 \pm 380$	$6480 \pm 420$	$29200 \pm 1900$	$27,600 \pm 1800$
Single top	$285 \pm 48$	$141 \pm 24$	$1830 \pm 310$	$860 \pm 150$
$W + bb/cc$	3$62 \pm 40$	$81 \pm 9$	$2640 \pm 290$	$506 \pm 56$
$W + c$	$174 \pm 47$	$8 \pm 2$	$1300 \pm 350$	$56 \pm 15$
$W + \hbox {light}$	$87 \pm 3$	$3.7 \pm 0.2$	$578 \pm 23$	$26 \pm 1$
$Z + \hbox {jets}$	$120 \pm 58$	$38 \pm 18$	$1190 \pm 570$	$310 \pm 150$
Diboson	$31 \pm 15$	$4 \pm 2$	$183 \pm 88$	$29 \pm 14$
Multijet	$228 \pm 68$	$38 \pm 11$	$2490 \pm 750$	$540 \pm 160$
Total expected	$7140 \pm 400$	$6790 \pm 420$	$39,400 \pm 2200$	$29,900 \pm 1800$
Data	6800	7056	37823	30644
(b) Muon+jets ($\mu$+jets) channel				
$t\bar{t}$	$7000 \pm 450$	$7640 \pm 490$	$35900 \pm 2300$	$33{,}500 \pm 2200$
Single top	$369 \pm 63$	$160 \pm 27$	$2110 \pm 360$	$980 \pm 170$
$W + bb/cc$	$473 \pm 52$	$117 \pm 13$	$3450 \pm 380$	$756 \pm 83$
$W + c$	$223 \pm 60$	$5 \pm 1$	$1540 \pm 420$	$63 \pm 17$
$W + \hbox {light}$	$96 \pm 4$	$1.8 \pm 0.1$	$797 \pm 32$	$40 \pm 2$
$Z + \hbox {jets}$	$74 \pm 36$	$16 \pm 8$	$610 \pm 290$	$159 \pm 76$
Diboson	$37 \pm 18$	$6 \pm 3$	$198 \pm 95$	$32 \pm 15$
Multijet	$195 \pm 59$	$34 \pm 10$	$1870 \pm 560$	$400 \pm 120$
Total expected	$8470 \pm 470$	$7980 \pm 490$	$46{,}400 \pm 2500$	$36{,}000 \pm 2200$
Data	8274	8193	46,275	36,471

### Reconstruction of the $t\bar{t}$ decay

The correct assignment of reconstructed jets to partons originating from a $t\bar{t}$ decay is important for this measurement. This is achieved using a likelihood-based method (Kinematic Likelihood Fitter, KLFitter [74]) which makes use of the Bayesian Analysis Toolkit [75]. KLFitter maps the four partons of the $t\bar{t}$ decay to four reconstructed jets using mass constraints on the top-quark mass $m_t$ and the *W*-boson mass $m_W$. In this analysis the four jets with the highest $p_{\text {T}}$ are used as input to KLFitter. A likelihood *L* is maximised for all resulting 24 permutations. For each permutation the likelihood is defined as

1 $$\begin{aligned} L&= \text {BW}(m_{q1q2q3}| m_t,\Gamma _{t})\cdot \text {BW}(m_{q1q2}| m_{W},\Gamma _{W})\nonumber \\&\quad \cdot \text {BW}(m_{q4\ell \nu }| m_t,\Gamma _{t})\cdot \text {BW}(m_{\ell \nu } | m_{W},\Gamma _{W}) \nonumber \\&\quad \cdot \prod \limits _{i=1}^4 W\left( E_{i}^{\text {meas}}|E_{i}\right) \cdot W\left( E_{\ell }^{\text {meas}}|E_{\ell }\right) \nonumber \\&\quad \cdot W\left( E_{x}^{\text {miss}}|p_{x}^{\nu }\right) \cdot W\left( E_{y}^{\text {miss}}|p_{y}^{\nu }\right) \,. \end{aligned}$$

The $W(E_{P}^{\text {meas}}|E_{P})$ are transfer functions, where $E_{P}^{\text {meas}}$ is the measured energy of the jet or lepton *P*, $E_{P}$ is the energy of the corresponding parton or lepton, and $p_{x}^{\nu }$ and $p_{y}^{\nu }$ are the momentum components of the neutrino $\nu$ in the transverse plane. These momentum components as well as the energies $E_P$ are free parameters of the likelihood maximisation. The component $p_{z}^{\nu }$ is initially calculated using a constraint on the *W*-boson mass $m_W^2 = (p_\nu + p_\ell )^2$ with the four-momenta $p_\nu$ and $p_\ell$ [74]. Transfer functions for electrons, muons, *b*-jets, light jets (including *c*-jets) and $E_{\text {T}}^{\text {miss}}$ are used. They are derived from simulated $t\bar{t}$ events using MC@NLO +Herwig  [29, 31, 32]. The decay products of the $t\bar{t}$ pair are uniquely matched to reconstructed particles to obtain a continuous function which describes the relative energy difference between a parton and a reconstructed jet or particle as a function of the parton energy. Parameterisations are derived for different $|\eta |$ regions. The $\text {BW}(m_{ij(k)}| m_{t/W},\Gamma _{t/W})$ terms represent Breit–Wigner functions which stand for the probability distribution of the reconstructed *W*-boson or top-quark mass given the assumed mass $m_{t/W}$ and a decay width $\Gamma _{t/W}$. Indices *q*1–*q*4 refer to the four quarks mapped to the reconstructed jets.

To exploit the presence of two *b*-quarks in a $t\bar{t}$ decay, kinematic information is complemented by *b*-tagging. To take it into account, the likelihood definition of Eq. (1) is extended and turned into an event probability which, for a given permutation *i*, is expressed as

$$\begin{aligned} P_{i}= \frac{L_i\prod _j p_{i,j}}{\sum _k L_k\prod _j p_{k,j}}\,. \end{aligned}$$

The $p_{i,j}$ contain the *b*-tagging efficiency or the mistag rate corresponding to the *b*-tagging working point, depending on the jet *j* flavour assigned by KLFitter and whether it is *b*-tagged or not. This factor is calculated for all jets *j* and multiplied by the likelihood $L_i$. KLFitter calculates the latter quantity for each permutation in the event according to Eq. (1). The permutation with the largest event probability determines the jet-to-parton assignment that is used to build the observables $m_{\ell b}$ and $\Delta R_{\text {min}}(j_b, j_l)$. In this analysis the mass parameters are set to $m_W=80.4~\text {GeV}$ and $m_t=172.5~\text {GeV}$ and the decay width parameters are fixed to $\Gamma _W = 2.1$ GeV and $\Gamma _t = 1.33$ GeV. The analysis uses KLFitter only to choose the best assignment of jets to partons and does not exploit the fitted four-momenta for the reconstructed particles. A variation of the $\Gamma _t$ parameter used in KLFitter was proven to have no impact on the reconstructed distributions and thus the extracted measured value of $\Gamma _t$.

**Fig. 3:**
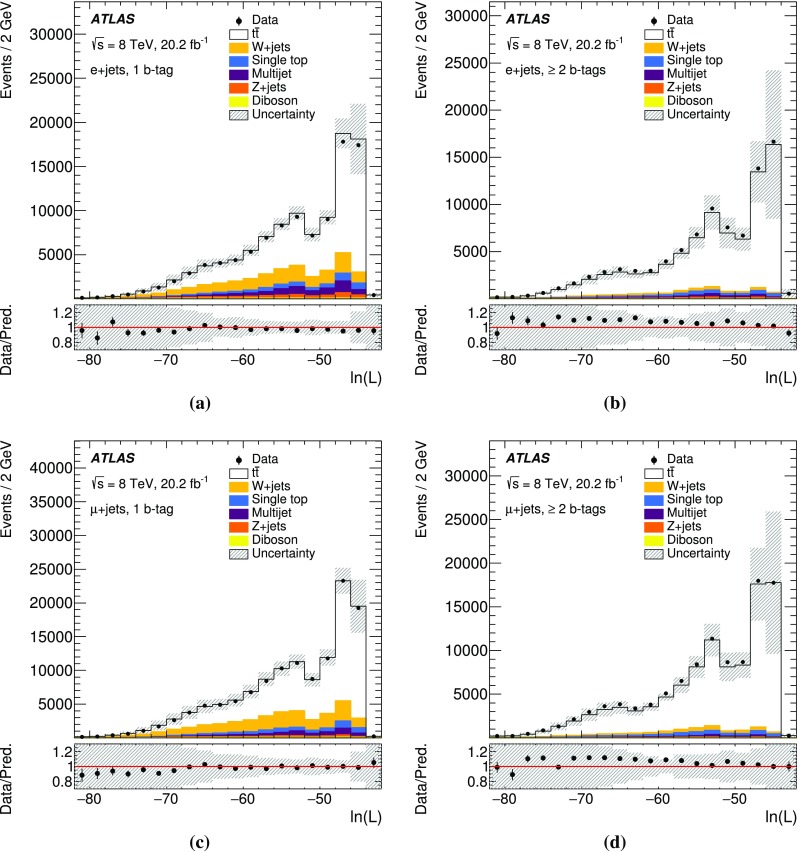
Distributions of the logarithm of the likelihood obtained from the event reconstruction algorithm for the selected **a**, **b** electron+jets and **c**, **d** muon+jets events with **a**, **c** exactly one and **b**, **d** at least two *b*-tagged jets. The hatched bands show the normalisation uncertainty in the signal and background contributions and the signal model systematic uncertainties. The first and last bins contain underflow and overflow events, respectively

Figure 3 shows distributions of the logarithm of the likelihood for different analysis regions. Fully matched $t\bar{t}$ events populate the high $\ln (L)$ region. Thus, a requirement of $\ln (L)> -50$ removes a significant fraction of the combinatorial background. However, both background events and $t\bar{t}$ events with partially correctly and incorrectly matched jets contribute to the full range of likelihood values. The double peak structure of the output is thus not related to a correct match of jets but caused by the migration of the events which are not matched correctly towards the higher values of the likelihood due to the fixed top quark mass requirement. The fraction of events where all four partons are matched correctly increases from 13 to 23% ( 17 to 31%) after applying this requirement to events with at least one (two) *b*-tagged jet(s). This selection also improves the purity of the sample by removing more background events than $t\bar{t}$ signal. The analysis does not rely on matching correctly all four jets. The observable $m_{\ell b}$, which provides most of the sensitivity to $\Gamma _t$, depends solely on the correct assignment of the *b*-jet from the semileptonically decaying top quark for which the reconstruction efficiency is 65% (75%) for events with at least one (two) *b*-tagged jet(s).

## Template fit

The decay width of the top quark is measured using a simultaneous template fit to distributions of two observables associated with the hadronic and semileptonic decay branches of $t\bar{t}$ events in the eight mutually exclusive analysis regions. The observables are $m_{\ell b}$, which is the reconstructed invariant mass of the *b*-jet of the semileptonically decaying top quark and the corresponding lepton, and $\Delta R_{\text {min}}(j_b, j_l)$, which is the angular distance between the *b*-jet $j_b$ and the closest light jet $j_l$, both originating from the hadronically decaying top quark. The choice of $m_{\ell b}$ is due to its good sensitivity to $\Gamma _t$ while being less sensitive to jet-related uncertainties compared to reconstructed masses of the hadronic decay branch. Despite the much lower sensitivity of $\Delta R_{\text {min}}(j_b, j_l)$ to $\Gamma _t$, it is beneficial to use it in the fit because it adds information from the hadronic top-quark decay branch and reduces leading jet-related and signal model systematic uncertainties in the combination with $m_{\ell b}$. Several other observables defined using the invariant mass of, or angles between, the $t\bar{t}$ decay products were tested but were found to be less suitable because of larger jet-related or signal model uncertainties.

**Fig. 4:**
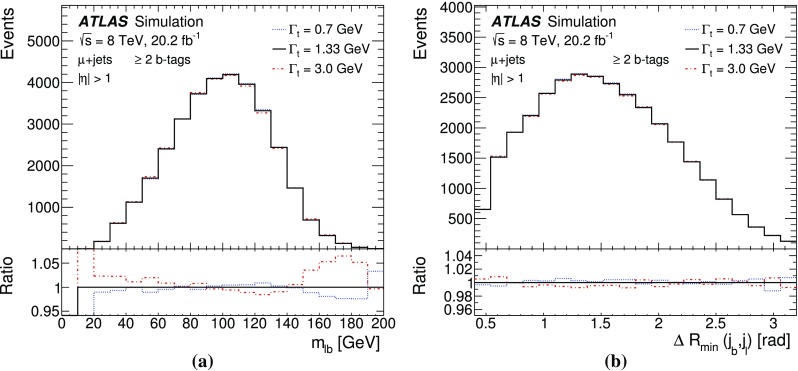
Templates for **a** the reconstructed invariant mass of the *b*-jet of the semileptonically decaying top quark and the corresponding lepton, $m_{\ell b}$, and **b** $\Delta R_{\text {min}}(j_b, j_l)$, the angular distance between the *b*-jet $j_b$ associated with the hadronic top quark and the closest light jet $j_l$ from the hadronically decaying *W* boson, in the range $0.7 \le \Gamma _t \le 3.0$ GeV in the muon+jets channel for events with at least two *b*-tags in the $|\eta | > 1$ region. The lower panel shows the ratio of the templates with varied $\Gamma _t$ to the nominal template generated for a decay width of $\Gamma _t = 1.33$ GeV

Signal templates are generated by reweighting events at parton-level to Breit–Wigner distributions with alternative top-quark decay-width hypotheses. A total of 54 templates for different values of $\Gamma _t$ are created: 50 templates cover the range $0.1< \Gamma _t < 5.0$ GeV in steps of $\Delta \Gamma = 0.1$ GeV. Four additional templates are created for $\Gamma _t = 0.01,~6,~7,~8$ GeV to take into account very small and very large width values. The top-quark decay width in the nominal MC signal sample is $\Gamma _t = 1.33$ GeV corresponding to the NLO calculation. The reweighting method was validated using a signal MC sample generated with $\Gamma _t = 3.0$ GeV by comparing top-quark mass distributions of this sample with top-quark mass distributions obtained from the reweighting procedure at parton level, and no significant differences were observed. The impact on the template distributions by varying the decay width in the range of 0.7–3.0 GeV is shown in Fig. 4.

The binned likelihood fit to data uses these signal templates for the $t\bar{t}$ contribution. Templates for all other processes, including single-top-quark production, are fixed. The effect on the result of using a fixed decay width in the single-top-quark template was found to be negligible. The number of expected events per bin *i* is given by

$$\begin{aligned} n_{i}= n_{\text {signal},i} + \sum \limits _{j=1}^{\text {B}} {n_{\mathrm {bkg},ji}}, \end{aligned}$$

where the index *j* runs over all backgrounds. The likelihood for an observable $\mathcal {O}$ is defined as follows:

2 $$\begin{aligned} \mathscr {L} (\mathcal {O}|\Gamma _t ) =&\prod \limits _{i=1}^{N_\text {bins}} \mathrm {Poisson} (n_{\mathrm {data},i} | n_{i}(\Gamma _t) )\nonumber \\&\cdot \prod \limits _{j=1}^{\text {B}}\frac{1}{\sqrt{2\pi }\sigma _{\mathrm {bkg},j}}\exp \left( {\frac{-(n_{\mathrm {bkg},j}-\hat{n}_{\mathrm {bkg},j})^2}{2\sigma ^{2}_{\mathrm {bkg},j}}} \right) , \end{aligned}$$

where $N_{\mathrm {bins}}$ is the number of bins in a template and $n_{\mathrm {data},i}$ is the number of data events in each bin *i*. The number of events from a background source *j*, $n_{\mathrm {bkg},j}$, is obtained from $n_{\mathrm {bkg},ji}$ by summing over all bins *i*. This number of background events varies in the fit but it is constrained by Gaussian terms where $\hat{n}_{\mathrm {bkg},j}$ is the expected number of background events for source *j* and $\sigma _{\mathrm {bkg},j}$ is its uncertainty. The total number of signal events is a free parameter of the fit. For each background source *j* only one fit parameter $n_{\mathrm {bkg},j}$ is used for all *b*-tag bins, lepton channels and $|\eta |$ regions except for the multijet background. For the latter, separate parameters are defined for each analysis region.

The uncertainties used as constraints in Eq. (2) on the *W*+jets background components normalisation originating from data-driven calibration (see Sect. 3) amount to 7% for $W+b\bar{b}$ and $W+c\bar{c}$, 25% for $W+c$, and 5% for *W*+light jets events. The uncertainty in the multijet background is taken from the matrix method and amounts to 30%. For the *Z*+jets and diboson events, a 4% theory uncertainty in the inclusive cross-section is applied together with a 24% uncertainty per additional jet added in quadrature, which covers the extrapolation to higher jet multiplicities based on MC studies, resulting in an uncertainty of 48% for events with four jets. The uncertainty in single-top-quark production amounts to 17% and considers the variation of initial- and final-state radiation in the *t*-channel MC samples and accounts for extra jets in single-top-quark events.

The fit is performed for 55 templates (54 obtained from the reweighting algorithm and the nominal one). The combined likelihood, defined as the product of two Poisson terms as given in Eq. (2), one for each observable, multiplied by the Gaussian constraints, is maximised for every value of $\Gamma _t$. The measured top-quark decay width is extracted from the minimum of a quadratic fit to the negative logarithm of the likelihood values. The fit method was validated using pseudo-experiments, and the correlation between the two observables was examined. In each pseudo-experiment the content of the bins of the $m_{\ell b}$ and $\Delta R_{\text {min}}(j_b, j_l)$ distributions are varied according to the Poisson and Gaussian distributions to take into account the expected number of events per bin and the background constraints, respectively. These pseudo-experiments are used for a linearity test and to produce pull distributions. The pull is defined as the difference between the fitted value $\Gamma _t$ and the input value divided by the estimated uncertainty of the fit result. No deviations from the expectation were found for various decay width values within $1.1< \Gamma _t < 4.0$ GeV. For smaller decay width values the pull width decreases since the $\Gamma _t$ distribution approaches a limit of 0 GeV. However, this does not affect the result and the fit method is stable and unbiased. Additional pseudo-experiments revealed that the small correlation between $m_{\ell b}$ and $\Delta R_{\text {min}}(j_b, j_l)$ of about (0.1–2.8)% does not affect the fit result. The observables are thus treated as independent.

## Systematic uncertainties

Systematic uncertainties affect the normalisation of signal and background and the shape of the distributions sensitive to the top-quark decay width. Individual sources of systematic uncertainty are considered uncorrelated and are summed in quadrature to determine the total uncertainty. Correlations of systematic uncertainties from the same source are fully maintained for all analysis regions. Pseudo-experiments are used to estimate the impact of the different sources of uncertainty according to the following procedure. For each source of systematic uncertainty, templates corresponding to the respective up and down variations are created. These variations consider shape and acceptance changes from the systematic uncertainty source under study. Pseudo-data sets are generated by imposing Poisson fluctuations and Gaussian fluctuations on the background contributions (to account for the Gaussian constraints) to each bin, as described in Sect. 5. Then the nominal and varied templates are used to perform a fit to pseudo-data. For each systematic variation 2000 of these pseudo-experiments were performed, and the differences between the means of the fitted-value distribution using the nominal templates and the up and down variations are quoted as the systematic uncertainty from this source. The systematic uncertainties in the measurement of the top-quark decay width are summarised in Table 2.

### Uncertainties in detector modelling

The systematic uncertainties arising from charged leptons are classified into several categories, related to the reconstruction and trigger efficiency, the identification and the lepton momentum scale and resolution. This leads to five (six) components of uncertainties associated with the electron (muon).

Jet-related uncertainties arise from the jet reconstruction efficiency, the jet vertex fraction requirement, the jet energy resolution (JER) and the jet energy scale. The JES and its uncertainties were derived by combining information from test-beam data, LHC collision data and simulation [65, 66]. The JES calibration is described in Sect. 4.1. The jet energy scale uncertainty is split into 26 $p_{\text {T}}$- and $\eta$-dependent sources, treated independently. It is the largest of the detector modelling uncertainties in this analysis.

The JER was evaluated separately for data and simulation using two in situ techniques [65], improved by additional in situ measurements using dijet, photon+jet or *Z*+jet processes. For low-$p_{\text {T}}$ jets, a significant contribution to the JER uncertainty comes from pile-up, measured as in Ref. [66]. The JER uncertainty consists of 11 components and represents an important uncertainty in this measurement. The systematic uncertainties originating from these components are summed in quadrature. The symmetrised difference is the quoted systematic uncertainty in the JER.

The per-jet efficiency to pass the JVF selection is evaluated in $Z(\rightarrow \ell ^+\ell ^-)$+1-jet events in data and simulation [67]. Motivated by this study, the uncertainty is estimated by changing the JVF requirement value, increasing and decreasing it by 0.1, and repeating the analysis using this modified value.

Energy scale and resolution correction uncertainties of both the leptons and jets are propagated into the calculation of $E_{\text {T}}^{\text {miss}}$. Contributions from energy deposits not associated with any jet and due to soft-jets (7 GeV $< p_\text {T}<$ 20 GeV) are also considered and treated as fully correlated with each other. A further $E_{\text {T}}^{\text {miss}}$ uncertainty accounts for mis-modeling of pileup energy deposits.

The jet-flavour-dependent efficiencies of the *b*-tagging algorithm are calibrated using data. The *b*-tagging efficiency is corrected to match the efficiency measured in the $t\bar{t}$ data events using the probability density function calibration method [70, 71] based on a combinatorial likelihood applied to a data sample of dileptonic $t\bar{t}$ events. The mistag rate for *c*-jets is measured using $D^*$ mesons, the one for light jets is measured using jets with impact parameters and secondary vertices consistent with a negative lifetime [69, 71]. Efficiencies for *b*- and *c*-jets are corrected in simulations by $p_\text {T}$-dependent scale factors. For light jets, these scale factors also depend on the pseudorapidity. Six independent sources of uncertainty affecting the *b*-tagging efficiency and four affecting the *c*-tagging efficiency are considered [70]. For the mistagging of light-quark jets, 12 uncertainties which depend on jet $p_{\text {T}}$ and $\eta$ [71] are considered.

### Uncertainties in background modelling

The uncertainties in the background normalisation are included as Gaussian constraints in the fit (see Eq. (2)) and thus contribute to the overall statistical uncertainty.

To estimate the uncertainty in the shape modelling of the *W*+jets background, each of its flavour components (*W*+$b\bar{b}/c\bar{c}$, *W*+*c* and *W*+light) is allowed to vary independently in the fit within its uncertainty, corresponding to the uncertainty in the calibration factors. The shape uncertainty of the *W*+jets contribution is retrieved by varying one component while fixing the other two to their respective normalisations, as given in Sect. 3.

Two simulated samples are compared to estimate the modelling uncertainty of single-top events. The baseline MC event generator for *Wt* production uses the diagram removal technique [76] to account for the overlap with $t\bar{t}$ events. This sample is compared to a sample generated with the inclusive diagram subtraction technique [76]. The difference is then symmetrised, i.e. the difference of the two-point comparison is taken as the uncertainty on both sides of the nominal result.

For the multijet background an uncertainty in the total yield of $\pm 30$% is assigned. Furthermore, two shape uncertainties are defined by varying the control samples used to obtain the efficiencies used in the matrix method to relate the two identification levels for objects considered as fake or non-prompt and prompt leptons, respectively.

The background yields estimated from MC simulation are affected by the luminosity uncertainty of 1.9% [20], which is propagated to the constraints on the background yields.

### Uncertainties in signal modelling

Several uncertainties affect the shape of the $t\bar{t}$ signal contributions. The uncertainties due to initial- and final-state radiation are determined using two dedicated Powheg+Pythia samples (see Sect. 3) generated with varied parameter values giving more or less radiation. Pseudo-data is created using each sample, and the largest variation of the top-quark decay width from the nominal is taken as an uncertainty and then symmetrised.

The Powheg MC event generator interfaced to Pythia is compared to Powheg interfaced with Herwig to estimate the uncertainty due to the parton shower and the hadronisation model. To estimate the uncertainty in the choice of the $t\bar{t}$ event generator, the full difference between Powheg and MC@NLO event generators, both interfaced with Herwig, is evaluated. This is the largest signal modelling uncertainty in this measurement. The uncertainty of the colour reconnection modelling is estimated by comparing the nominal $t\bar{t}$ sample to a Powheg sample interfaced with Pythia with the Perugia parameter tune “P2012IoCR“ [27] for colour reconnection. This tune has a slightly lower colour reconnection strength than the default tune, which affects the corresponding colour strings, and is combined with a slightly modified colour reconnection algorithm. The uncertainty in the underlying-event modelling is determined by comparing the nominal $t\bar{t}$ sample with a Powheg sample interfaced with Pythia employing the Perugia parameter tune “P2012mpiHI” [27] for multiparton interactions. This tune increases the number of multi-parton interaction (MPI) scatterings, which leads to an increase in MPI minijets. This is realised by a larger $\alpha _S$ value associated with the MPI. The uncertainties due to these four sources are taken as the difference between the nominal and the varied sample and symmetrised, i.e. the full difference is taken as the positive and negative uncertainty.

Following the PDF4LHC [77] recommendations, three different PDF sets are compared using a reweighting technique for the signal $t\bar{t}$ MC sample to estimate the uncertainty due to the PDF set choice: CT10 NLO (nominal PDF set) [25], MSTW 2008 68% CL NLO [52] and NNPDF 2.3 NLO [78]. Each PDF set has a different prescription for using its error sets to evaluate the uncertainty: the CT10 set uses a symmetric Hessian matrix, the MSTW set uses an asymmetric Hessian matrix and the NNPDF set uses a standard deviation for the uncertainty calculation. For the three PDF sets, the variations for all different PDF parameters are evaluated within the corresponding set. Half of the width of the largest deviation from nominal among all three sets is taken as the PDF uncertainty.

### NLO and off-shell effects in the top-quark decay

The $t\bar{t}$ MC simulation utilised to extract the decay width uses NLO matrix elements for top-quark pair production and LO matrix elements with approximate implementation of finite-width and interference effects for the decay of the top quarks. A theoretical study [79] performed in the $e\mu$ decay channel of the $t\bar{t}$ system indicates that taking into account off-shell effects, which include the contributions from $t\bar{t}$ and *Wt* single-top production as well as their interference, is important for the precision measurements of top-quark properties. However, there is no MC implementation yet of NLO decay and off-shell effects for the lepton+jets final state. The potential impact of ignoring these effects was tested in two different ways. First, the parton-level $m_{\ell b}$ distribution of a sum of $t\bar{t}$ and *Wt* single-top contributions without these effects taken into account was reweighted to the $m_{\ell b}$ distribution provided by the authors of Ref. [79] which corresponds to the $WWb\bar{b}$ calculation at NLO. Second, the measurement was repeated requiring $m_{\ell b} < 150$ $\text {GeV}$, limiting the analysis to the region where these effects are expected to be suppressed according to Ref. [79]. Both cross-checks yield a difference in the measured top-quark decay width of less than 0.5 GeV. For more precise future measurements, it would be beneficial to have an MC simulation providing an NLO description of the top-quark decay accounting for off-shell effects.

**Table 2:** Summary of systematic uncertainties in the top-quark decay width measurement

Source	Uncertainty (GeV)
Detector model	
Electron	$^{+0.14}_{-0.07}$
Muon	$^{+0.11}_{-0.06}$
Jet energy scale	$^{+0.42}_{-0.30}$
Jet energy resolution	$\pm 0.27$
Jet vertex fraction	$^{+0.13}_{-0.03}$
Jet reconstruction efficiency	$\pm 0.03$
Missing transverse momentum	$\pm 0.01$
*b*-Tagging	$^{+0.32}_{-0.24}$
Signal model	
ME event generator	$\pm 0.41$
Colour reconnection	$\pm 0.19$
Underlying event	$\pm 0.11$
Radiation	$\pm 0.07$
PDF	$\pm 0.06$
PS/hadronisation	$\pm 0.05$
Background model	
Multijet	$^{+0.04}_{-0.00}$
*W*+jets	$\pm 0.02$
Single top	$< 0.01$
Template statistical uncertainty	$\pm 0.07$
Luminosity	$^{+0.03}_{-0.00}$
Total systematic uncertainty	$^{+0.79}_{-0.68}$

### Template statistical uncertainty

To estimate the systematic uncertainty arising from the limited MC sample size used to produce the templates, the content of each bin of the signal and background templates is varied within its statistical uncertainty and a fit to the nominal distribution is repeated. The MC statistical uncertainty is derived with and without taking into account the correlations between the templates and both estimates yield consistent results. The standard deviation of the distribution of top-quark decay width values obtained from the fits with the fluctuated templates is quoted as the systematic effect from the template statistical uncertainty.

## Result

**Fig. 5:**
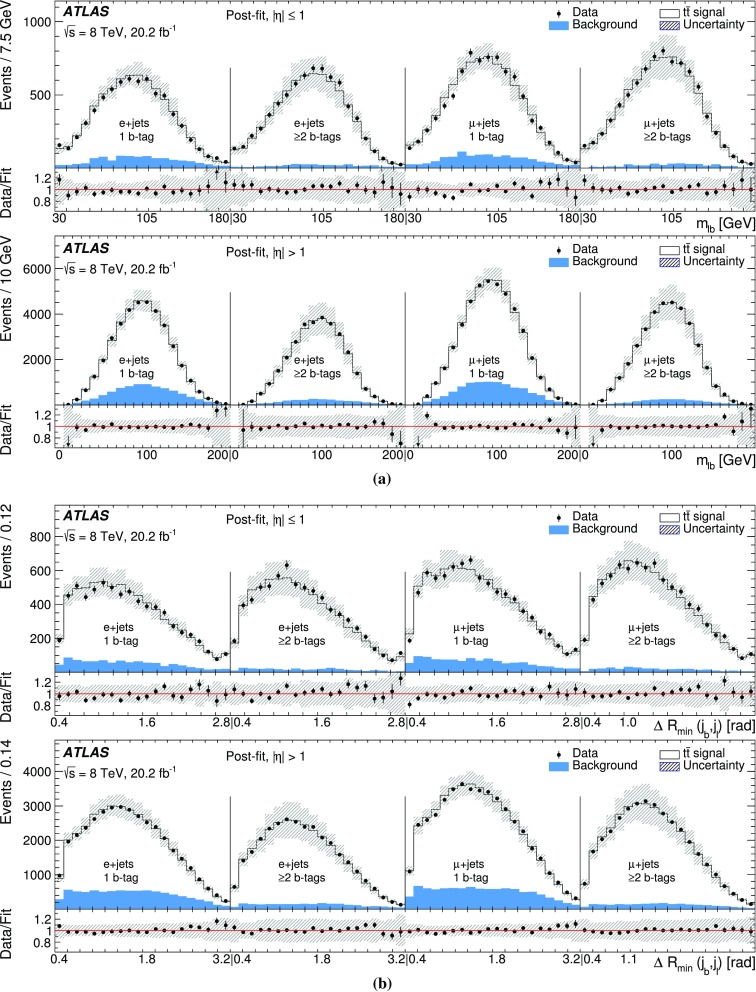
Post-fit distributions based on the best-fit templates for **a** $m_{\ell b}$ and **b** $\Delta R_{\text {min}}(j_b, j_l)$. The background contributions are combined. The lower panel shows the ratio of data to post-fit sum of $t\bar{t}$ signal and background. The eight analysis regions corresponding to different *b*-tag multiplicity and jet pseudorapidity are shown. The vertical lines show the boundaries between the binned variables in different lepton and *b*-tag regions. The hatched band shows the total uncertainty. The systematic uncertainties are calculated bin-by-bin from the systematic variations by adding differences in quadrature. Then, statistical and systematic uncertainties are added in quadrature to obtain the quoted total uncertainty

The binned likelihood template fit is applied to the data using the concatenated distributions of $m_{\ell b}$ and $\Delta R_{\text {min}}(j_b, j_l)$ in the eight analysis regions. Figure 5 shows post-fit distributions for $m_{\ell b}$ and $\Delta R_{\text {min}}(j_b, j_l)$. The post-fit yields of the $t\bar{t}$ signal and each background contribution are summarised in Table 3.

**Table 3:** Post-fit yields of the $t\bar{t}$ signal and background contributions. The yields represent the sum of the number of events in each of the eight analysis regions. Only the normalisation uncertainties are shown

Sample	Post-fit yields
$t\bar{t}$	$156{,}360 \pm 750$
Single top	$5700 \pm 930$
$W + \hbox {bb/cc}$	$7060 \pm 510$
$W + \hbox {c}$	$1650 \pm 550$
$W + \hbox {light}$	$1603 \pm \,\,65$
$Z + \hbox {jets}$	$2770 \pm 710$
Diboson	$320 \pm 240$
Multijet	$6070 \pm 380$
Total	$181,600 \pm 1700$
Data	181,536

**Fig. 6:**
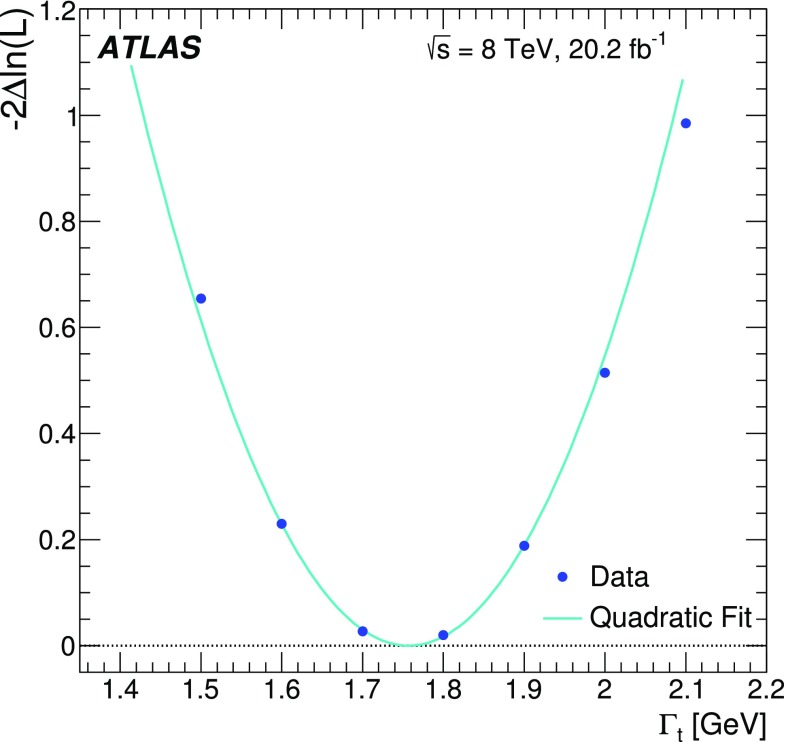
Twice the negative logarithm of the likelihood obtained from the binned likelihood template fit to data. A quadratic fit is performed around the minimum

The likelihood curve obtained from the fit can be seen in Fig. 6 together with a quadratic fit to the likelihood points. The statistical uncertainty, which includes contributions from the data statistics and the uncertainties in the backgrounds normalisation, is extracted from the likelihood curve’s width at $-2\Delta \ln (\mathscr {L})=1$ around the minimum. The likelihood values are shifted so that the minimum corresponds to $-2\Delta \ln (\mathscr {L}) = 0$.

The measured decay width for a top-quark mass of 172.5 GeV is

$$\begin{aligned} \Gamma _t = 1.76 \pm 0.33~(\text {stat.})~ ^{+0.79}_{-0.68}~(\text {syst.})~\text {GeV} = 1.76^{+0.86}_{-0.76}~\text {GeV}, \end{aligned}$$

in good agreement with the SM prediction of 1.322 GeV [9]. A consistency check was performed by repeating the measurement in the individual *b*-tag regions and confirms that the results are consistent with the measured value. A fit based only on the observable $m_{\ell b}$ leads to a total uncertainty which is about 0.3 GeV larger.

In comparison to the previous direct top-quark decay width measurement in Ref. [18], the total uncertainty of this measurement is smaller by a factor of around two. However, this result is still less precise than indirect measurements and, thus, alternative (BSM) models discussed in Sect. 1 cannot be ruled out with the current sensitivity.

The impact of the assumed top-quark mass on the decay width measurement is estimated by varying the mass around the nominal value of $m_t=172.5$ GeV. Changing the top-quark mass by $\pm 0.5$ GeV leads to a shift in the measured top-quark decay width of up to around 0.2 GeV.

## Conclusion

A direct measurement of the decay width of the top quark exploiting $t\bar{t}$ events in the lepton+jets channel was performed using data taken in proton–proton collisions at $\sqrt{s} = 8$ TeV corresponding to an integrated luminosity of 20.2 fb$^{-1}$ recorded by the ATLAS detector at the LHC. The decay width of the top quark is extracted using a binned likelihood template fit to data based on two observables related to the hadronic and the semileptonic decay branches of the $t\bar{t}$ pair. The top-quark decay width is measured to be $\Gamma _t = 1.76 \pm 0.33~(\text {stat.})~ ^{+0.79}_{-0.68}~(\text {syst.})~\text {GeV}$ for $m_t = 172.5$ GeV, which is in a good agreement with SM predictions.

## Footnotes

1 ATLAS uses a right-handed coordinate system with its origin at the nominal interaction point (IP) in the centre of the detector and the *z* axis along the beam pipe. The *x* axis points from the IP to the centre of the LHC ring, and the *y* axis points upwards. Cylindrical coordinates $(r,\phi )$ are used in the transverse plane, $\phi$ being the azimuthal angle around the *z* axis. The pseudorapidity is defined in terms of the polar angle $\theta$ as $\eta = -\ln \tan (\theta /2)$. Angular distance is measured in units of $\Delta R \equiv \sqrt{(\Delta \eta )^{2} + (\Delta \phi )^{2}}$.

2 The primary vertex is required to have at least four associated tracks with $p_{\text {T}} > 400$ MeV. If more than one vertex fulfils this criterion, the one with the largest sum of squared transverse momenta from its associated tracks is defined to be the primary vertex.

3 The jet vertex faction (JVF [68]) is the ratio of the sum of the $p_{\text {T}}$ of tracks associated with the jet and the primary vertex to the sum of $p_{\text {T}}$ of all tracks associated with the jet, while the $p_{\text {T}}$ of the involved tracks needs to exceed 1 GeV. Hence, this selection ensures that at least 50% of the sum of the $p_{\text {T}}$ of tracks associated with the jet belongs to tracks compatible with originating from the primary vertex.
